# Fibroblast Activation Protein Inhibitor (FAPI)-Based Theranostics

**DOI:** 10.3390/ph18040522

**Published:** 2025-04-03

**Authors:** William Serumula, Venesen Pillay, Bawinile Hadebe, Mariza Vorster

**Affiliations:** Department of Nuclear Medicine, School of Health Sciences, University of KwaZulu-Natal, Durban 4058, South Africa; william.tebogo88@gmail.com (W.S.); venesen.pillay@gmail.com (V.P.); bawzahadebe@yahoo.com (B.H.)

**Keywords:** FAPI, PET, theranostics

## Abstract

Fibroblast activation protein (FAP) is a serine protease selectively expressed in cancer-associated fibroblasts (CAFs), fibrotic tissues, and areas of active tissue remodeling, making it an attractive target for diagnostic imaging across a spectrum of disease. FAP inhibitors (FAPIs) labeled with PET tracers have rapidly advanced as a novel imaging modality with broad clinical applications that offers several advantages, including rapid tumor accumulation, low background uptake, and high tumor-to-background ratios. In oncology, FAPI PET has demonstrated excellent performance in visualizing a wide range of malignancies, including those with low glycolytic activity, such as pancreatic cancer, cholangiocarcinoma, and certain sarcomas. Its high sensitivity and specificity for the stromal component enables improved tumor delineation, staging, and response assessment. Additionally, the potential to guide theranostic approaches, where the same tracer can be labeled with therapeutic radionuclides, positions FAPI as a key player in precision oncology. Beyond oncology, FAPI PET has shown promise in imaging conditions characterized by fibrotic and inflammatory processes. In the cardiovascular field, FAPI PET imaging is being investigated for its ability to detect myocardial fibrosis and active cardiac remodeling, crucial in conditions like heart failure, post-myocardial infarction remodeling, and hypertrophic cardiomyopathy. This review highlights the expanding clinical applications of FAPI-based PET imaging across oncology, inflammation, and cardiovascular disease. While the current data are promising, further large-scale studies and multicenter trials are essential to validate these findings and establish standardized protocols. The versatility and broad applicability of FAPI PET underscore its potential as a transformative tool in precision medicine.

## 1. Introduction to FAPI Theranostics

**Figure 1:**
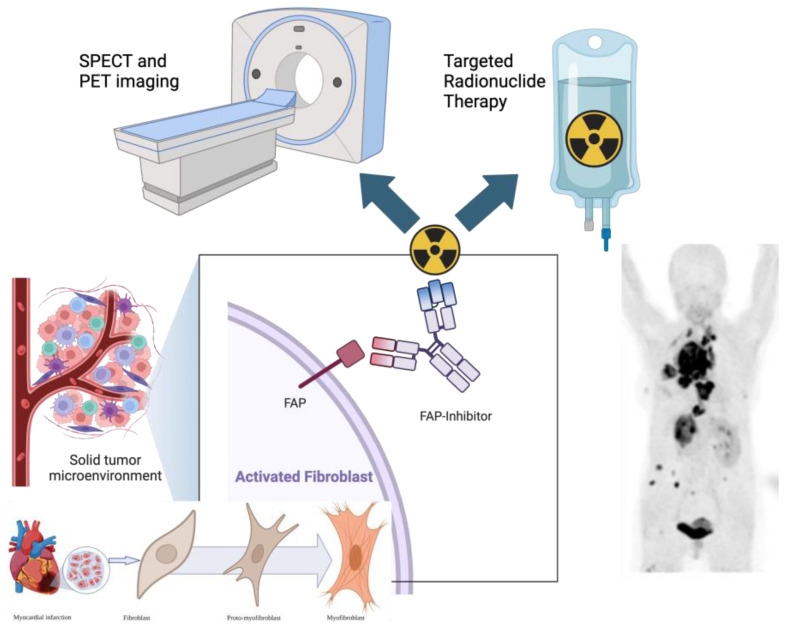
The Tumor Microenvironment (TME): cancer-associated fibroblasts express fibroblast activation protein (FAP), which serves as a versatile target for PET imaging and therapeutic approaches with FAP inhibitors (image created in BioRender).

The tumor microenvironment (TME) is a multifaceted and complex tumor structure with a dynamic framework, which plays a significant role in tumor cells’ survival through a pro-tumorigenic signaling pathway [1,2]. This has prompted drug development research to re-focus and shift from the tumor to the TME for novel drug development [3,4]. Furthermore, this has gained interest in molecular imaging applications and potential therapies. The discovery of fibroblast activation protein inhibitor positron emission tomography (FAPI PET) has led to an explosion in the number of clinical applications, not just in oncology, but also related to cardiovascular disease and infective and inflammatory processes (See Figure 1).

Fibroblast activation protein (FAP) is a membrane-bound serine protease highly expressed in cancer-associated fibroblasts (CAFs) within the tumor microenvironment (TME). It possesses both gelatinase and dipeptidyl peptidase activity, enabling the cleavage of multiple substrates, including type I collagen and dipeptides. FAP plays a pivotal role in extracellular matrix remodeling, which is crucial for tissue repair and wound healing [5,6,7].

Positron emission tomography/computed tomography (PET/CT) is widely used in oncology for tumor detection, staging, and monitoring treatment responses across various malignancies [8]. The most commonly utilized radiotracer, ^18^F-fluorodeoxyglucose (^18^F-FDG), exhibits a high sensitivity in cancer detection. However, it has notable limitations, including an inability to reliably differentiate malignancy from infection or inflammation, a high physiological uptake in the brain, gastrointestinal tract (GIT), and genitourinary tract (GUT), and the requirement for a nearby cyclotron for production [9,10].

Recent studies suggest that FAPI-based PET imaging may outperform ^18^F-FDG PET/CT in detecting tumors, particularly in malignancies with a low glucose metabolism [11,12,13]. Additionally, FAPI PET holds potential for diagnosing benign fibrotic and inflammatory conditions [14]. While most FAPI tracers remain in developmental stages, emerging data suggest that FAPI PET/CT will play an important clinical role, despite its absence from current oncology guidelines [11].

Systematic reviews and meta-analyses have demonstrated that FAPI PET/CT exhibits a high sensitivity in detecting primary tumors, lymph node metastases, and distant metastases. This makes it an effective tool for cancer staging and treatment monitoring by specifically targeting the TME with FAPI-based radiopharmaceuticals [6,15,16].

Loktev et al. first introduced FAPI-based radiotracers, leading to a surge of studies evaluating their diagnostic accuracy and clinical utility [7,17,18]. These studies consistently reported a high tumor-to-background contrast and superior accuracy in detecting various cancers. However, boron pro-alanine-FAPIs and radio-FAPIs were initially described by Poplawski et al. in 2013 [19]. Comparative studies assessing FAPI PET/CT, ^18^F-FDG-PET/CT, and other imaging modalities have been conducted to evaluate their performance in cancer detection, staging, and response assessment [20,21]. Four recent meta-analyses further confirmed that FAPI PET/CT demonstrates a higher sensitivity than ^18^F-FDG PET/CT in detecting primary tumors and metastases, particularly in low-glucose-avid cancers [22,23,24,25]. Notably, tumors such as pancreatic cancer, breast cancer, sarcomas, gastrointestinal cancers, and gynecological malignancies exhibit a high FAP expression in the tumor stroma, making them ideal candidates for FAPI-based imaging [6,15,20].

The growing evidence surrounding FAPI PET/CT has broadened its applications beyond oncology. Studies indicate that FAPI PET imaging surpasses ^18^F-FDG PET/CT in multiple cancer types and may also prove valuable in non-oncologic conditions [11]. This suggests that FAPI PET holds significant promise in detecting tumors where ^18^F-FDG PET/CT exhibits a limited sensitivity and specificity [11]. However, FAPI PET has certain limitations, particularly its uptake in inflammatory and fibrotic processes, which may lead to false-positive findings [11]. A key knowledge gap remains in determining whether FAPI PET/CT can reliably distinguish between benign and malignant lesions, as FAPI demonstrates avidity for both [26,27,28,29,30]. Despite these challenges, FAPI PET is a promising imaging modality with significant clinical potential. Further research is needed to develop alternative FAPI tracers that offer an improved specificity, cost-effectiveness, and broader clinical utility [31,32,33]. To maximize its clinical impact, large-scale trials with diverse patient cohorts are required to validate its role across different malignancies.

### FAPI-Based Radioligands

In addition, the integration of FAPI-targeted radioligands for image-guided therapy could pave the way for new treatment strategies in both oncologic and non-oncologic settings [26,34,35]. The potential therapeutic benefits of FAP inhibition further underscore the need for larger prospective studies to define its role in cancer treatment. Direct comparisons between FAPI PET/CT and ^18^F-FDG PET/CT remain critical to determining their relative advantages in clinical practice [13,36,37,38,39]. A systematic review and meta-analysis by Wass et al. demonstrated that FAPI PET/CT exhibited a higher sensitivity than ^18^F-FDG PET/CT in detecting primary tumors, nodal involvement, and metastatic lesions. However, ^18^F-FDG PET/CT showed a higher specificity for primary tumors, underscoring the need for further validation of FAPI PET/CT’s diagnostic performance [40].

One of the most significant advantages of Gallium-68 FAP-based tracers is their potential for theranostic applications. By replacing Gallium-68 (a diagnostic isotope) with therapeutic radionuclides (e.g., Lutetium-177, Actinium-225, or Yttrium-90), FAPI-based imaging can seamlessly transition into targeted radionuclide therapy, a stepwise approach known as “theranostics”. This dual capability provides a distinct advantage over FDG-PET-based imaging.

The first theranostic application of FAP-targeted radiotracers was reported using Yttrium-90-labeled FAPI-04 (^90^Y-FAPI-04) in a patient with metastatic breast cancer, leading to significant symptom relief and reduced analgesic requirements [41,42]. Subsequent studies have demonstrated successful therapeutic applications of Yttrium-90- and Samarium-153-labeled FAPI-46 [34,43,44]. Preclinical and clinical studies investigating Actinium-225-labeled FAPI-04 further highlight the growing potential of FAPI-based theranostics [44].

Table 1 provides an overview of the most clinically relevant FAPI ligands that have been evaluated for diagnostic imaging and theranostic applications.

## 2. Fibroblast Activation Protein in Cancer Imaging

Fibroblast activation protein (FAP) is highly overexpressed on the membrane of cancer-associated fibroblasts (CAFs) in approximately 90% of epithelial-derived tumors. An elevated FAP expression is associated with a poor prognosis and adverse treatment outcomes across various cancers. In addition to their presence in malignant tumors, CAFs are found in areas of tissue damage, remodeling, and chronic inflammation, as well as in some benign conditions. In contrast, FAP expression is minimal or absent in normal tissues, making it a promising target for molecular imaging and therapeutic applications.

This selective overexpression has led to the development of FAP-specific inhibitors (FAPIs) labeled with positron-emitting radionuclides, enabling PET imaging to visualize and quantify FAP expression in tumors. FAPIs are small-molecule inhibitors that selectively bind to FAP, allowing for the highly specific imaging of tumors with significant stromal involvement. This precision enhances the detection of both primary and metastatic lesions across a wide range of cancer types.

While ^18^F-FDG PET/CT remains a widely used imaging modality for cancer diagnosis, its non-specific glucose uptake often results in false-positive findings. Additionally, ^18^F-FDG PET/CT has limitations in detecting tumors with low glycolytic activity, leading to false-negative results, particularly in lobular breast cancer, mucinous-type cancers, and non-seminomatous germ cell tumors (NSGCTs).

Recently, there has been increasing interest in FAPI-based PET imaging due to its favorable biodistribution and more convenient imaging protocols. Unlike ^18^F-FDG, FAPI-based imaging does not require extensive patient preparation, and scans can begin as early as 10 min post-injection, significantly reducing scan time and patient burden. Furthermore, FAPI tracers exhibit a lower background uptake in key organs such as the heart, liver, and brain, enhancing their ability to detect pathology in these regions with improved contrast. Beyond its role in imaging, FAP expression is closely linked to tumor aggressiveness and prognosis, with higher expression levels often correlating with poorer patient outcomes. This association further supports the expanding role of FAPI-based PET imaging in both diagnostic and prognostic evaluations, making it a powerful tool for precision oncology.

## 3. The Potential Role of FAPI PET Imaging in Specific Cancers

### 3.1. CNS Malignancies

A 2024 study by Liu et al. evaluated 40 lesions in 25 patients with brain tumors and found that ^68^Ga-FAPI PET/CT outperformed ^18^F-FDG PET/CT in lesion detection (75% vs. 62.5%), particularly in identifying metastatic brain tumors (63.6% vs. 45.5%) [46]. Similarly, a recent study by Hua et al. [47] compared ^68^Ga-FAPi-04 and ^18^F-FET (fluoride-18 fluoroethyl-L-tyrosine) in 22 adult patients with intracranial mass lesions. Their findings revealed heterogeneous FAP expression across intracranial tumors, with more malignant tumors—such as brain metastases, glioblastoma, and medulloblastoma—exhibiting a higher ^68^Ga-FAPi-04 uptake compared to less aggressive tumors [47].

Overall, ^68^Ga-FAPI PET/CT demonstrates a superior performance compared to ^18^F-FDG PET/CT in detecting systemic brain metastases, particularly in cases where MRI is contraindicated (e.g., due to metal implants or claustrophobia). A key advantage of FAPI imaging is its higher sensitivity for detecting brain lesions due to negligible background activity, unlike ^18^F-FDG, which shows intense physiological uptake in the brain. Additionally, ^68^Ga-FAPI PET/CT may serve as a more cost-effective alternative in specific clinical settings, further expanding its potential in neuro-oncology.

### 3.2. Head and Neck Cancer

Zhao et al. [48] reported that ^68^Ga-FAPI-04 PET/CT demonstrated a significantly higher uptake than ^18^F-FDG PET/CT in patients with primary nasopharyngeal carcinoma, leading to an improved detection of regional lymph node involvement, bone metastases, and visceral metastases. This superior uptake resulted in the identification of more positive lesions, particularly in challenging areas such as the skull base and intracranial region.

In cases of head and neck cancer of unknown primary origin where ^18^F-FDG PET/CT yielded negative findings, ^68^Ga-FAPI-04 PET/CT successfully identified the primary tumor in 7 of 18 patients (39%). Additionally, ^68^Ga-FAPI-04 PET/CT appeared superior to ^18^F-FDG PET/CT in the preoperative detection of metastatic lymph nodes in head and neck squamous cell carcinoma and oral squamous cell carcinoma, highlighting its potential as a valuable imaging modality in these malignancies [48].

### 3.3. Thyroid Cancer

^18^F-FDG PET/CT is recommended by the American Thyroid Association guidelines for detecting tumor recurrence and metastases in radioactive iodine (RAI)-refractory differentiated thyroid carcinomas (DTCs). Additionally, it is utilized for aggressive thyroid cancer variants, including Hürthle cell carcinoma, insular carcinoma, poorly differentiated thyroid carcinoma (PDTC), and anaplastic thyroid carcinoma (ATC) [49]. However, its sensitivity ranges from 68.8% to 82%, and its clinical utility is often limited by high false-positive rates, primarily due to inflammatory lymphadenopathy. Furthermore, a false-negative rate of 8–21.1% has been reported in patients with TENIS syndrome, further complicating the management of metastatic DTCs.

A meta-analysis by Guglielmo et al. [50] demonstrated that the overall diagnostic performance of FAPI-labeled PET/CT was comparable to ^18^F-FDG PET/CT. However, FAPI imaging exhibited higher SUVmax values in sites of local recurrence and lymph node metastases, particularly in patients with BRAFV600E mutations [50]. Notably, TSH, thyroglobulin (Tg), and Tg antibody (Tg-Ab) levels did not influence SUVmax values, indicating that FAPI imaging remains unaffected by these factors.

Ballal et al. [51] further demonstrated the superiority of ^68^Ga-DOTA.SA.FAPI PET/CT in detecting metastases in lymph nodes, the liver, brain, and bowel, while also reporting lower false-positive and false-negative rates compared to ^18^F-FDG PET/CT. Regarding lung metastases, ^68^Ga-DOTA.SA.FAPI PET/CT exhibited a higher detection accuracy than ^18^F-FDG PET imaging [51]. Similarly, Nourbakhsh et al. found that FAPI-based PET/CT identified additional lung metastases, leading to upstaging in 20% of patients with lung metastases [52].

In medullary thyroid cancer (MTC), Ballal et al. [51] demonstrated that ^68^Ga-DOTA.SA.FAPI PET/CT and [^68^Ga]Ga-DOTA-NOC PET/CT exhibited a comparable sensitivity in detecting primary tumors, metastatic lymph nodes, and brain and pleural metastases. However, ^68^Ga-DOTA.SA.FAPI PET/CT showed significantly higher detection rates for lung nodules, liver, and bone metastases compared to ^68^Ga-DOTA-NOC. Additionally, ^68^Ga-DOTA-FAPI-04 demonstrated higher uptake values and tumor-to-background ratios (TBRs) in all evaluated metastatic lesions [53].

**Figure 2:**
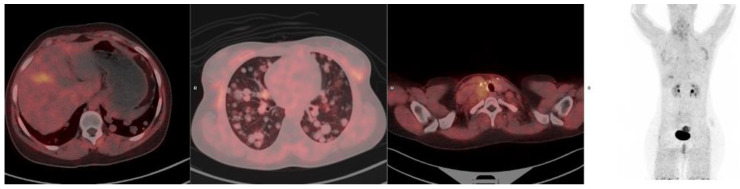
^68^Ga-FAPI-46 PET/CT images of a 19-year-old female with metastatic papillary thyroid cancer (follicular variant). In addition to the cervical and lymph node metastases, false positive uptake is seen in the gallbladder on ^68^Ga-FAPI-46.

Despite its advantages, FAPI uptake is not entirely specific for malignancies, as it can also accumulate in non-cancerous conditions, including fibrosis and autoimmune diseases. FAPI-based radiotracers primarily reflect fibrotic activity rather than active inflammation. In thyroid disease, an increased FAPI uptake has been observed in follicular thyroid adenomas with fibrosis, as well as in conditions such as calcification, thyroiditis, myelofibrosis, reactive lymph nodes, arthritis, subcutaneous fibroma, and Graves’ ophthalmopathy [53]. (Figure 2 demonstrates false-positive uptake in the gallbladder of a patient imaged with ^68^Ga-FAPI PET for metastatic follicular thyroid cancer.)

At present, ^18^F-FDG PET/CT remains the preferred imaging modality for restaging RAI-refractory thyroid cancer, given its widespread availability, cost-effectiveness, and standardized quantification techniques. However, FAPI PET/CT may serve as a complementary tool for prognostication and selecting candidates for FAP-targeted therapies. Additionally, in medullary thyroid cancer, FAPI-based PET/CT may outperform somatostatin receptor imaging, offering a promising alternative for disease evaluation and management.

### 3.4. Breast Cancer

Several studies have demonstrated the superior sensitivity of FAPI PET/CT compared to ^18^F-FDG PET/CT in detecting both primary breast lesions and metastatic disease [5,11,54,55]. Mori et al. [11] reported that ^68^Ga-FAPI PET/CT provided a better visualization of primary breast cancer lesions than ^18^F-FDG PET/CT. In a lesion-based analysis, the primary tumor detection rates were 100% (50/50) for ^68^Ga-FAPI PET/CT and 96% (48/50) for ^18^F-FDG PET/CT.

Additionally, the SUVmax values on ^68^Ga-FAPI PET/CT correlated positively with the pathological grade and clinical stage of primary breast tumors. A key advantage of FAPI imaging is its ability to accumulate in breast cancer subtypes with low metabolic activity, such as lobular and luminal A breast cancers, where ^18^F-FDG PET/CT has a reduced sensitivity [56,57].

**Figure 3:**
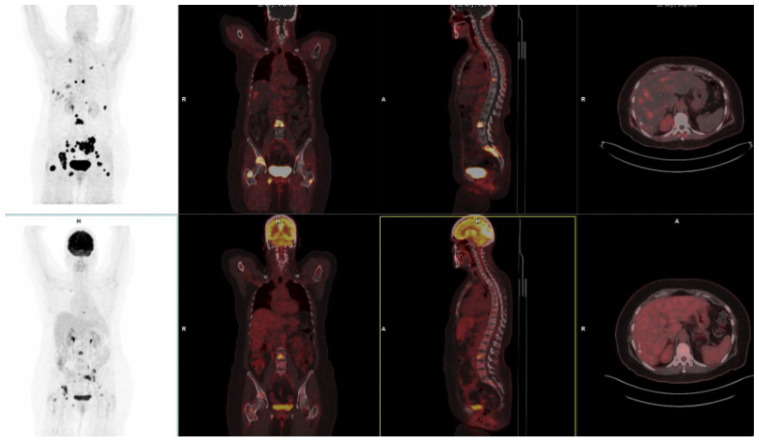
^68^Ga-FAPI-46 (**top panel**) and ^18^F-FDG PET/CT (**bottom panel**) images of a 60-year-old female with luminal A breast cancer (ER + ve, PR + ve, Ki-67 60%), demonstrating superior detection of visceral metastasis with FAPI compared to FDG. ^68^Ga-FAPI (**top panel**) shows multiple foci of intense tracer accumulation in the liver, multiple ribs, vertebrae, and pelvis. Corresponding ^18^F-FDG images (**bottom panel**) show less intense and fewer areas of tracer accumulation compared to ^68^Ga-FAPI.

Beyond detecting primary lesions, ^68^Ga-FAPI PET/CT identified additional lesions with a lower background activity and greater uptake in lymph node, liver, lung, and bone metastases in breast cancer patients [58]. Figure 3 highlights the superior detection of breast cancer metastases on FAPI PET/CT compared to FDG PET/CT. Furthermore, ^68^Ga-FAPI PET/CT enables early response assessment during and after treatment, providing a valuable tool for therapy monitoring [59].

### 3.5. Limitations and Clinical Considerations of FAPI PET in Breast Cancer

Despite its advantages, FAPI PET imaging in breast cancer has limitations, particularly in specificity, as FAPI uptake has been observed in various benign conditions with inflammatory components. These include benign tumors, fibrosis, granulomas, scarring, wounds, and other degenerative or inflammatory diseases [55]. This non-specific uptake may lead to false-positive findings. Additionally, benign bone cysts have been reported to exhibit an increased ^68^Ga-FAPI uptake, potentially mimicking bone metastases in lobular breast cancer, further complicating interpretation.

### 3.6. Clinical Relevance and Theranostic Potential

Overall, FAPI PET appears superior to ^18^F-FDG PET/CT in breast cancer imaging, with studies demonstrating its ability to upstage disease and influence management decisions in up to 17% of patients. Furthermore, FAPI PET has an emerging theranostic potential in breast cancer, offering a dual role in both diagnosis and targeted radionuclide therapy. However, FAPI uptake can occasionally occur in non-malignant breast lesions, including accessory breast tissues, fibroadenomas, and benign lymphoid tissues [58]. As a result, incidental breast uptake on FAPI PET/CT should be interpreted with caution, necessitating correlation with clinical and histopathological findings to avoid misdiagnosis.

### 3.7. Lung Cancer

Surgery remains the primary treatment for stage I–IIIA non-small-cell lung cancer (NSCLC). Lymph node (LN) metastasis is a key prognostic factor in NSCLC, as the complete resection of metastatic LNs plays a crucial role in improving overall survival and disease-free survival [60,61]. FAP is upregulated in lung cancer, but its expression varies by subtype. NSCLC can express FAP in up to 100% of cases, whereas small-cell lung cancer (SCLC) and large-cell neuroendocrine carcinoma (LCNC) exhibit FAP expression in up to 67% of cases [59].

A meta-analysis by Yang et al. demonstrated that FAPI PET/CT exhibits a higher sensitivity than ^18^F-FDG PET/CT. ^18^F-FDG PET/CT achieved a 99% sensitivity for the diagnostic evaluation of initial lung cancer lesions, compared to 98% for FAPI PET/CT [59]. Additionally, a meta-analysis of seven studies involving 409 lung cancer patients showed that FAPI PET/CT provided a superior sensitivity and specificity in detecting lymph node metastases compared to ^18^F-FDG PET/CT [60]. FAPI PET/CT outperformed ^18^F-FDG PET/CT in identifying positive lymph nodes [61] and distant metastases, owing to its higher tumor-to-background contrast. Additionally, FAPI PET/CT has demonstrated a greater sensitivity than ^18^F-FDG in detecting brain, hepatic, pleural, and bone metastases [61].

FAPI PET/CT has emerged as a more precise imaging modality for preoperative staging and surgical candidate selection in NSCLC, owing to its superior sensitivity in detecting metastatic involvement. This enhanced accuracy not only aids in refining treatment strategies, but also plays a crucial role in optimizing surgical decision making, potentially improving patient outcomes.

### 3.8. Esophageal Cancer

**Figure 4:**
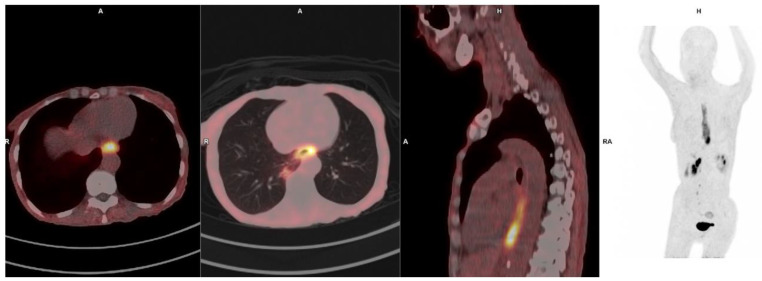
65-year-old female with SCC of the distal esophagus; ^68^Ga-FAPI-46 PET/CT demonstrates intense tracer accumulation in an extensive lesion extending from T2–T10 with no distant metastasis.

Recent studies have highlighted the superior performance of FAPI PET imaging over ^18^F-FDG PET/CT in detecting esophageal cancer. Zhao et al. [62] reported that FAPI PET demonstrated higher primary tumor SUVs and a greater detection sensitivity than ^18^F-FDG PET, particularly in the distal third of the esophagus [63,64]. Furthermore, Ristau et al. suggested that FAPI PET may play a crucial role in tumor delineation for radiotherapy planning, given its higher tumor-to-background ratio (TBR) compared to ^18^F-FDG PET [64]. Additionally, Wegen et al. reported that FAPI PET imaging led to a change in management in 25% of 32 esophageal cancer patients who underwent evaluation with both FDG and FAPI PET/CT [65]. Figure 4 demonstrates the intense uptake noted in a patient with an esophagus carcinoma imaged with ^68^Ga-FAPI-46 PET/CT.

### 3.9. Gastric Cancer

In gastric cancer, the sensitivity of FDG is limited by the normal tracer bio-distribution in the gastric wall [66]. In a meta-analysis by Xie et al., FAPI was superior to FDG for the detection of primary gastric cancer lesions, as well as metastatic deposits in the lymph nodes and peritoneum, with no significant difference observed for the detection of metastasis to the liver, bone, and ovary [67]. For the detection of gastro-intestinal cancer recurrence, FAPI was superior to FDG. In a meta-analysis by Wu et al. the pooled sensitivities of ^68^Ga-FAPI-04 PET and ^18^F-FDG PET were 100% and 59% respectively [68]. The results of the pooled specificities of ^68^Ga-FAPI-04 PET and ^18^F-FDG PET for gastrointestinal cancer recurrence were 66% and 46% respectively [68].

### 3.10. Pancreatic Cancer

In pancreatic cancer, FDG has a limited specificity due to positivity in non-malignant lesions such as chronic pancreatitis. In addition, false-negative findings are seen in 10% of pancreatic cancer patients [67]. FAPI is also taken up in pancreatitis, however, it has been demonstrated that dual-time-point (3 h delayed) imaging could potentially differentiate pancreatic cancer from pancreatitis, thus increasing specificity [69]. FAPI-targeted PET has been reported to be superior to FDG PET and CECT for the staging of pancreatic cancer, showing a superior detection of the primary and lung metastases [69,70]. In a meta-analysis, Tang et al. reported a FAPI PET/CT pooled sensitivity of 100% for primary pancreatic cancer lesions, whereas FDG PET/CT performed slightly worse in diagnosing primary pancreatic cancer lesions, with a pooled sensitivity of 89%. FAPI PET imaging had a significantly higher sensitivity for identifying lymph node metastasis than FDG PET imaging (62.4% vs. 37.3%) [70].

### 3.11. Hepatocellular Carcinoma

The utility of ^18^F-FDG PET/CT in detecting liver tumors is limited due to the presence of glucose-6-phosphatase in hepatocytes. This enzyme dephosphorylates glucose-6-phosphate and FDG-6-phosphate, allowing ^18^F-FDG to exit the cell, reducing intracellular tracer retention. Additionally, well to moderately differentiated hepatocellular carcinoma (HCC) exhibits lower expressions of GLUT-1 and GLUT-2, further limiting FDG uptake. Combined with a high physiological background activity in the liver, these factors reduce tumor-to-background ratios (TBRs) and impair the detectability of hepatic malignancies on ^18^F-FDG PET/CT [71].

A meta-analysis by Singh et al. reported that FAPI PET/CT demonstrated a superior sensitivity (94.3%) compared to ^18^F-FDG PET/CT (56.1%) in detecting primary liver tumors. However, ^18^F-FDG PET/CT exhibited a higher pooled specificity (96.4%) compared to FAPI PET/CT (89.3%), suggesting potential limitations of FAPI imaging in differentiating malignant and benign liver lesions [71].

A major limitation of FAPI PET/CT in liver imaging is its lower specificity, primarily due to false-positive findings. Manuppella et al. reported that ^68^Ga-FAPI PET/CT detected more liver lesions in cases of primary hepatic inflammatory myofibroblastoma compared to ^18^F-FDG PET/CT. Other causes of false-positive uptake in the liver include arteriovenous malformations, biliary obstruction, liver abscesses, focal nodular hyperplasia, and inflammatory liver nodules [72]. Additionally, in patients with liver cirrhosis, a diffusely increased background FAPI uptake has been observed, potentially masking malignant lesions and leading to false-negative findings. This highlights the importance of cautious interpretation when assessing FAPI PET/CT scans in liver pathology.

Overall, FAPI PET/CT demonstrates a superior performance over ^18^F-FDG PET/CT in the detection of liver malignancies, benefiting from a lower physiological liver uptake and improved tumor-to-background contrast. However, correlative imaging (e.g., MRI or CT) remains essential to differentiate malignant from benign hepatic lesions, minimizing diagnostic pitfalls due to false-positive findings.

### 3.12. Colorectal Cancer

^18^F-FDG PET/CT is widely used in the management of colorectal cancer (CRC); however, its effectiveness is limited by physiological bowel uptake, a low specificity, and a poor sensitivity in mucinous and signet-ring cell carcinomas. Additionally, ^18^F-FDG uptake in some adenomas can lead to false-positive findings, reducing its specificity.

A meta-analysis by Zhuang et al. reported that ^68^Ga-FAPI-04 PET/CT demonstrated a higher detection rate for lymph node and peritoneal metastases compared to ^18^F-FDG PET/CT. However, the detection rates for skeletal metastases were similar for both tracers [73].

In a study by Lin et al., which compared ^18^F-FDG and FAPI PET/CT in 61 colorectal cancer patients, FAPI PET/CT was superior for detecting primary tumors and liver metastases. Notably, FAPI PET imaging resulted in a change in clinical management for 21% of patients, with upstaging in 16.4% and downstaging in 8.2% [74].

### 3.13. FAPI PET/CT in Gynecological Malignancies

A pooled analysis showed that ^68^Ga-FAPI PET/MR and ^18^F-FDG PET/CT had a high sensitivity for lymph node metastases, with detection rates of 98% and 85%, respectively. In peritoneal metastases from ovarian cancer, FAPI PET/CT demonstrated a sensitivity of 98% (95% CI = 0.93–1) compared to 71% (95% CI = 0.55–0.86) for ^18^F-FDG PET/CT [75]. Physiological ^68^Ga-FAPI-04 uptake in the uterus was found to be significantly higher than ^18^F-FDG uptake, likely due to active fibroblast presence, which may obscure lesions [75,76]. Dendl et al. demonstrated that FAPI PET/CT provided superior lesion detection in gynecological malignancies, with a better tumor-to-background ratio (TBR) compared to ^18^F-FDG PET/CT [76]. In cervical cancer, FAPI PET/CT exhibited a superior detection of liver, bone, and lymph node metastases compared to ^18^F-FDG PET/CT, highlighting its potential role in staging and disease monitoring [77].

### 3.14. FAPI PET/CT in Soft-Tissue Sarcomas and Gastrointestinal Stromal Tumors (GISTs)

In patients with recurrent soft-tissue sarcomas, ^68^Ga-FAPI-04 PET/CT detected more lesions than ^18^F-FDG PET/CT, demonstrating a superior sensitivity, specificity, and diagnostic accuracy, leading to upstaging in 4 of 45 patients (8.9%) [78]. Additionally, ^18^F-FAPI-42 PET/CT has been reported to detect more tumor lesions (85/106) than ^18^F-FDG PET/CT (57/106) in cases of metastatic or recurrent gastrointestinal stromal tumors (GISTs). This difference was particularly notable in liver metastases, where FAPI PET/CT detected 42/48 lesions compared to only 6/48 detected on ^18^F-FDG PET/CT [79]. These findings suggest that FAPI PET/CT could serve as a valuable imaging tool for monitoring soft-tissue sarcoma and GIST recurrence.

### 3.15. FAPI PET/CT in Genitourinary Malignancies

FAPI PET/CT has demonstrated a superior tumor-to-background contrast compared to ^18^F-FDG PET/CT in bladder cancer, allowing for an improved detection of metastatic lesions [80,81].

In metastatic castration-resistant prostate cancer (mCRPC), FAPI PET/CT may play a role in identifying tumor heterogeneity and detecting PSMA-negative lesions that do not respond to PSMA-targeted radionuclide therapy, potentially guiding alternative treatment approaches [80]. Findings in renal cell carcinoma (RCC) have been inconsistent, with some studies reporting a superior detectability of RCC using FAPI PET/CT, while others have found no significant advantage [81].

The role of FAPI PET/CT in genitourinary cancers remains under investigation. While PSMA PET remains the preferred imaging modality for prostate cancer, FAPI PET may be valuable for detecting PSMA-negative disease, allowing for potential alternative therapeutic strategies targeting FAP expression. In bladder cancer, FAPI PET/CT holds promise as an imaging modality due to its higher tumor-to-background contrast compared to ^18^F-FDG PET/CT. However, further research is needed to fully establish its diagnostic and prognostic utility.

**Figure 5:**
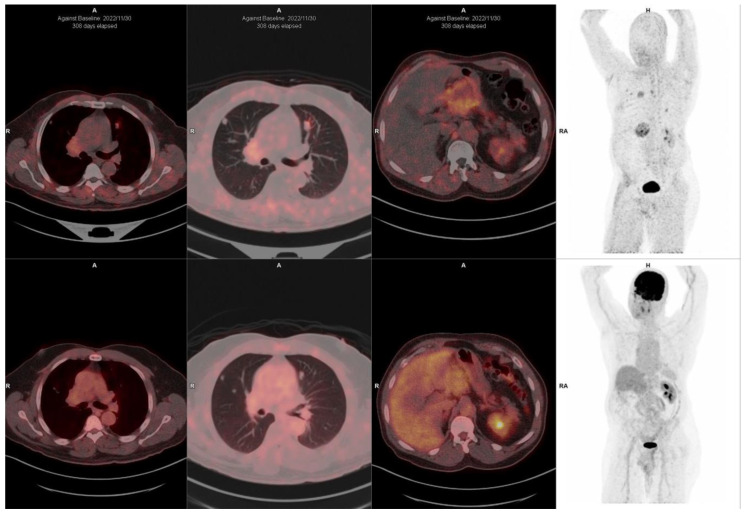
65-year-old female with renal cell carcinoma post right nephrectomy who presented with a mass in the region of the head of pancreas. ^68^Ga-FAPI-46 (**upper panel**) shows avidity in the head of pancreas mass, right hilar lymph node, and the lung compared to ^18^F-FDG (**lower panel**). These lesions are better seen on the FAPI-46 PET images due to the better tumor-to-background ratio.

Figure 5 illustrates the superior visualization of a pancreatic head mass, right hilar lymph node, and lung lesions using FAPI PET/CT compared to ^18^F-FDG PET/CT in a patient with metastatic renal cell carcinoma.

## 4. Summary of the Role of FAP-Based Imaging in Oncology

Fibroblast activation protein inhibitor (FAPI) PET imaging has emerged as a valuable tool in oncology, providing a superior tumor delineation compared to ^18^F-FDG PET across various malignancies. Unlike ^18^F-FDG, which relies on glucose metabolism and is often limited by a high physiological uptake in the brain and inflammatory tissues, FAPI PET specifically targets fibroblast activation protein (FAP), which is overexpressed in cancer-associated fibroblasts (CAFs) within the tumor microenvironment. This selective uptake results in a high tumor-to-background contrast, making FAPI PET particularly effective for detecting primary and metastatic lesions, as well as for staging, re-staging, and treatment response assessment.

The broad applicability of FAPI PET/CT extends to multiple cancer types, including head and neck, pancreatic, hepatocellular, breast, colorectal cancers, and sarcomas. Its favorable biodistribution and rapid clearance offer significant advantages in the detection of small lesions, particularly in cases of carcinoma of unknown origin (CUP), and it has shown potential in upgrading tumor staging and influencing clinical management decisions.

As evidence supporting its clinical utility continues to grow, FAPI PET is poised to complement or even surpass ^18^F-FDG PET in select oncologic applications. Additionally, FAPI imaging is gaining recognition for its emerging role in theranostic applications, offering potential for both diagnostic imaging and targeted radionuclide therapy in precision oncology.

## 5. FAPI PET in Cardiovascular Disease

Fibrosis is one of the key healing responses to injury. In the myocardium, it is especially important in the short term for maintaining structural integrity following an acute insult, e.g., myocardial infarction (MI). Conversely, should the process become dysregulated, fibrosis can lead to adverse remodeling, increased rigidity, and progressive dysfunction, with eventual cardiac failure with the risk of fatal arrythmias [82]. Myocardial fibrosis underlies almost every cardiomyopathic condition, making it a key target for imaging and therapeutic interventions. Further, an improved understanding of the timing of appropriate and inappropriate fibrotic responses after an acute insult (e.g., MI) will be key in preventing the transition to heart failure and sudden cardiac death whilst leaving the protective scar intact [83,84]).

While the non-invasive identification of fibrosis is important, current cardiac magnetic resonance (CMR) methods, including late gadolinium enhancement (LGE), are useful for detecting established fibrosis (reversible and diffuse, and irreversible replacement type). The shortcomings of such techniques include a reduced spatial resolution to assess fibrosis in valves and atherosclerotic plaques, being unable to identify the stage of fibrinogenesis activity, and being unable to distinguish between active and inactive or burnt-out disease [85]. Radiolabeled FAPI has been identified to address these shortcomings by identifying activated fibroblasts and active fibrosis, with the benefit of tracking fibroblast activity over time and in response to treatment [86].

Activated fibroblasts, groups of cells in the connective tissue that synthesize collagen and other components of the extracellular matrix (ECM), are the key cells driving fibrinogenesis in the myocardium and other organs [84]. The advantage of FAPI tracers is that they target the more exclusive endopeptidase activity of fibroblast activation protein (FAP), making them highly specific for this protein; furthermore, after FAP binding, FAPIs become rapidly and almost completely internalized into FAP+ fibroblasts, with minimal release into the surrounding tissue [7].

The emerging literature advocating support for the usage of FAPI tracers in imaging various cardiovascular diseases is growing. The relatively common areas under investigation include MI, myocardial disease, atherosclerosis, inflammatory vascular disease, and arrhythmias [84,87,88].

### 5.1. Myocardial Infarction

The inflammatory pathways associated with acute MI have varying effects on structural parameters and clinical outcomes. Following acute inflammation, cardiac repair is characterized by activated fibroblasts, along with the aggregation of structural proteins shaping the extracellular matrix (ECM). While this phenomenon can be cardioprotective, overzealous activation triggers an excessive accumulation of these proteins, ultimately leading to fibrosis, stiffness, and finally, heart failure [89,90]. FAPI tracers have been identified as surrogate markers to help determine the optimal time point to initiate anti-inflammatory therapies [88].

Varasteh et al. demonstrated peak FAPI uptake in the myocardium 6 days post-MI in animal models, with a higher uptake in the border regions and minimal uptake in the remote myocardium up to 11 days [91]. Diekmann et al. demonstrated a good concordance between FAPI uptake and an infarcted myocardium in clinical studies. They demonstrated in a cohort of 35 patients up to 11 days post-MI that the volume of FAP uptake positively correlated with LGE on CMR and exceeded the area of perfusion on myocardial perfusion imaging (MPI). The volume of FAPI uptake correlated with a fall in left ventricular ejection fraction (LVEF) at a median of 140 days post-MI in 14 of the 35 patients [92]. Similar results were observed in smaller studies, where FAPI uptake also extended beyond the infarct zone and correlated with LV remodeling and LVEF [92,93].

### 5.2. Myocardial Diseases

Of note, remodeling of the extracellular matrix (ECM) is a shared feature not only in individuals suffering from acute MI, but also in other cardiovascular diseases, including, but not limited to cardiomyopathies and conditions that lead to heart failure, aortic stenosis (AS), arrhythmogenesis, or atherosclerosis [84,88].

Clinical models demonstrate findings similar to animal models, showing an increased FAPI uptake in pressure-overload conditions, heart failure with reduced ejection fraction (HFrEF), heart failure with preserved ejection fraction (HFpEF) [94,95,96], and anthracycline-induced cardiotoxicity [96].

FAPI uptake did not necessarily correspond to areas of reduced perfusion on MPI in non-ischemic cardiomyopathy or with established scarring in LGE in chronic thromboembolic pulmonary hypertension and hypertrophic cardiomyopathy. FAPI uptake did, however, correlate positively with the 5-year sudden cardiac death score in hypertrophic cardiomyopathy, demonstrating a potential prognostic role in this group [95,97].

In AS, cardiac fibrosis has been advocated to play a role of eminent significance in patients scheduled for transcatheter aortic valve replacement (TAVR) [88]. These patients demonstrated an increased FAPI uptake, with its volume correlating with markers of heart failure, where the increased fibrotic load was tightly linked to less favorable cardiac functional outcomes and higher rates of heart failure. This indicates a potential for its use in prognostication [98,99].

### 5.3. Atherosclerosis

Fibrosis is also involved in atherosclerosis, mainly by balancing the inflammatory response (triggering plaque rupture) and profibrotic activity in chronic settings (chiefly mediating stability). Imaging FAPI tracers with hybrid camera systems has the advantage of providing information on fibroblast activity in the vessels, while CT can assist with identifying the plaque burden [88,100,101].

Kosmala et al. found that FAPI uptake was seen in only about half of calcified arterial lesions [102], with Wu et al. demonstrating the intensity of FAPI uptake correlating inversely with the extent of calcification [103]. In larger arterial vessels such as the aorta and femoral arteries, FAPI has been useful to characterize plaques with thin fibrous caps that may be prone to rupture compared to their stable calcific equivalent [102,103].

### 5.4. Other Cardiovascular Conditions

There are now numerous case reports describing myocardial FAPI uptake in several other conditions, including sarcoidosis and hypertensive heart disease [84]. The vascular uptake of FAPI has been demonstrated in patients with Takayasu’s arteritis, giant cell arteritis, and IgG4-related disease, being visualized in predominantly large- and medium-sized vessels [104,105]. Loganath et al. concluded following their review that, due to the high sensitivity of FAPI PET, it can be used to identify fibroblast activation in thin-walled structures where reliable fibrosis imaging has previously been challenging, particularly in the right ventricle and cardiac atria [84]. The detection of right ventricular fibroblast activity might be of particular value in patients with arrhythmogenic cardiomyopathy, pulmonary hypertension, and congenital heart disease. Similarly, with the atria, FAPI PET holds promise in improving our understanding of atrial cardiomyopathy and the triggers of atrial fibrillation.

## 6. Summary of the Potential Role of FAP-Based Imaging in Cardiovascular Disease

Fibroblast activation protein inhibitor (FAPI) PET imaging has emerged as a transformative tool in cardiovascular disease (CVD), offering unique insights into fibrotic processes that were previously difficult to assess. Its ability to non-invasively visualize activated fibroblasts in real time allows for a more nuanced understanding of fibrogenesis in various cardiovascular conditions.

In myocardial infarction (MI), FAPI PET has shown promise in detecting active fibrosis during critical post-injury periods, aiding in the identification of optimal therapeutic windows for anti-inflammatory and anti-fibrotic interventions. Studies have demonstrated strong correlations between FAPI uptake and adverse cardiac remodeling, left ventricular dysfunction, and future heart failure risk, positioning FAPI PET as a valuable prognostic tool.

Beyond MI, FAPI imaging has broadened its applications to non-ischemic cardiomyopathies, heart failure with preserved or reduced ejection fraction, aortic stenosis, and arrhythmogenic conditions. Its ability to detect fibroblast activity even in the absence of overt scarring—as observed with conventional modalities like late gadolinium enhancement (LGE) on CMR—highlights its superiority in identifying early fibrotic changes and potential arrhythmogenic substrates.

In atherosclerosis, FAPI PET offers novel insights by differentiating between stable and high-risk plaques based on fibroblast activity, potentially improving risk stratification for plaque rupture. Additionally, FAPI uptake has been noted in inflammatory vascular diseases and thin-walled cardiac structures, such as the right ventricle and atria, where conventional imaging struggles to accurately assess fibrotic burden.

The growing body of literature underscores FAPI PET’s potential in both the diagnostic and prognostic realms, with applications spanning from MI to inflammatory vascular diseases. Its specificity for activated fibroblasts not only enhances diagnostic precision, but also opens up new avenues for targeted image-guided therapies. However, further large-scale studies are essential to standardize its clinical use, refine interpretation criteria, and fully integrate FAPI PET into cardiovascular imaging algorithms.

In conclusion, FAPI PET holds significant promise in revolutionizing the imaging of cardiovascular diseases, providing a deeper understanding of fibrotic pathophysiology, improving patient stratification, and guiding therapeutic decisions. As research continues to evolve, FAPI imaging could become a cornerstone in the management of fibrotic cardiovascular conditions.

## 7. The Role of FAPI PET Imaging in Inflammatory and Infectious Conditions

### 7.1. Role in Inflammation Imaging

Fibroblast activation protein inhibitor (FAPI) PET imaging has demonstrated significant potential in evaluating a range of inflammatory conditions. By targeting fibroblast activation protein (FAP), which is overexpressed in activated fibroblasts during tissue remodeling and fibrosis, FAPI PET enables the precise imaging of fibrotic and inflammatory processes.

### 7.2. Immune-Mediated Inflammatory Diseases (IMIDs)

Lartey et al. [106] highlighted the efficacy of FAPI PET/CT in detecting fibrosis across diverse IMIDs, including interstitial lung diseases, systemic sclerosis, and inflammatory bowel disease. FAPI PET outperformed conventional imaging modalities in detecting early fibrotic changes, showing a strong correlation with disease severity and providing a valuable tool for disease staging and treatment monitoring [106]. The study emphasized FAPI PET’s ability to identify subclinical fibrosis, potentially allowing for earlier interventions and improved patient outcomes.

### 7.3. Systemic Vasculitis

Zhong et al. [107] performed a comparative study between ^18^F-FAPI-42 PET/CT and ^18^F-FDG PET/CT in systemic vasculitis, establishing FAPI PET’s superior lesion detection rate and better correlation with inflammatory markers. The study underscored FAPI PET’s utility in disease monitoring and its potential to guide treatment decisions [107]. The enhanced visualization of vascular inflammation could lead to more precise risk stratification and management.

Sollini et al. [22] conducted an early systematic review and meta-analysis in 2021, assessing the clinical utility of FAPI PET imaging. While it primarily focused on oncological applications, the review identified FAPI PET’s potential in non-oncological conditions, such as systemic sclerosis and IgG4-related disease, with a high sensitivity and specificity reported for detecting fibrotic and inflammatory lesions [22]. The review emphasized the need for standardized imaging protocols to enhance diagnostic accuracy.

### 7.4. Inflammatory Arthritis

Schmidkonz et al. [108] explored the utility of FAPI PET/CT in inflammatory arthritis, especially rheumatoid arthritis (RA). The imaging modality effectively identified activated fibroblast-like synoviocytes, which are crucial in RA pathogenesis, providing a superior visualization of synovial inflammation compared to FDG PET, thus aiding in early diagnosis and monitoring [108]. This capability is particularly beneficial in evaluating treatment response and tailoring therapeutic strategies.

### 7.5. Interstitial Lung Disease (ILD) and Fibrosis Imaging

Hotta et al. [109] explored the utility of ^68^Ga-FAPI-46 PET/CT in ILD, demonstrating a positive correlation with the immunohistochemical FAP expression score. This suggests FAPI PET as a potential imaging biomarker for ILD. The non-invasive nature of FAPI PET makes it an attractive option for the longitudinal monitoring of fibrotic lung diseases, potentially guiding antifibrotic therapy.

### 7.6. Role in Infection Imaging

Beyond inflammatory conditions, FAPI PET imaging has shown promise in detecting and evaluating various infectious diseases (most notably TB, COVID-19, Aspergillosis, and peri-prosthetic joint infections), leveraging its ability to identify fibroblast activation in infected tissues.

### 7.7. Tuberculosis (TB)

Studies such as those by Hao et al. [110] have demonstrated the efficacy of ^68^Ga-DOTA-FAPI-04 PET/CT in identifying tuberculous lesions. FAPI PET showed intense tracer uptake in disseminated TB, outperforming ^18^F-FDG PET/CT, particularly in detecting lesions obscured by a high physiological FDG uptake [110]. Similarly, Liu et al. [111] reported strong FAPI uptake in pulmonary TB lesions, although highlighting potential false positives due to overlapping uptake patterns with malignancies [111]. These findings suggest FAPI PET’s potential role in improving TB diagnosis, especially in complex cases.

### 7.8. Post-COVID-19 Lung Changes

In the context of COVID-19, Sviridenko et al. [112] used ^68^Ga-FAPI-46 PET/CT to evaluate persistent pulmonary abnormalities post-infection. FAPI PET effectively highlighted fibrotic lesions that were not visible on FDG PET, suggesting its role in detecting ongoing fibrotic repair rather than active inflammation [112]. Musameh et al. [113] further validated these findings, demonstrating the ability of FAPI PET to differentiate active fibrosis from stable scar tissue in long-term post-COVID-19 lung disease [113]. This differentiation is critical in guiding therapeutic decisions and predicting long-term outcomes.

### 7.9. Aspergillosis and Other Fungal Infections

Kullik et al. [114] showcased the application of dual-tracer PET/CT using ^18^F-FDG and ^68^Ga-FAPI in differentiating *Aspergillus fumigatus* infection from tumor recurrence. FAPI PET imaging provided crucial insights into complex infectious scenarios, aiding in accurate diagnosis [4]. The ability to distinguish infectious from malignant lesions can significantly impact patient management and treatment planning [114].

### 7.10. Periprosthetic Joint Infections (PJI)

Wang et al. [115] assessed ^68^Ga-DOTA-FAPI-04 PET/CT in diagnosing PJI, demonstrating its ability to differentiate between infection and aseptic loosening with a high sensitivity and specificity. This capability is particularly valuable in orthopedic settings, where accurate diagnosis can influence surgical decisions and postoperative care [115].

## 8. Summary of the Potential Role of FAP-Based Imaging in Infection- and Inflammation Imaging

Two systematic reviews recently performed to evaluate the role of FAPI-based PET in infection and inflammation imaging provides us with the highest levels of evidence in this setting to date.

In 2023, Bentestuen et al. [116] performed an expedited systematic review focusing on non-malignant FAPI-avid PET/CT findings. The review included 108 studies, revealing frequent FAPI uptake in benign inflammatory and fibrotic tissues, notably in arterial plaques, arthritis, and tuberculosis. This highlighted FAPI PET’s high sensitivity for fibroblast activity, but also emphasized the need for careful interpretation to avoid false positives [116]. The review underscored the importance of clinical correlation and multimodal imaging.

Albano et al. [117] provided a comprehensive systematic review targeting FAPI PET’s application in infectious and inflammatory diseases. Analyzing 21 studies with 1046 patients, the review demonstrated consistently high detection rates for FAPI PET in lung interstitial diseases, bone and joint disorders, IgG4-related disease, and Crohn’s disease. FAPI PET was frequently superior to ^18^F-FDG PET/CT, particularly in chronic inflammation and tissue remodeling contexts [117]. The review highlighted FAPI PET’s potential role in guiding personalized treatment strategies.

To summarize, FAPI PET imaging has emerged as a versatile and sensitive tool for evaluating a variety of inflammatory and infectious diseases. Its ability to detect activated fibroblasts involved in fibrosis and tissue remodeling allows for superior lesion detection and improved differentiation between benign and malignant lesions. Systematic reviews and comparative studies have established FAPI PET’s diagnostic accuracy, often surpassing conventional FDG PET/CT, particularly in fibrotic and inflammatory conditions. As research progresses, FAPI PET is poised to play an increasingly central role in diagnostic imaging and disease management across a spectrum of inflammatory and infectious diseases.

## 9. Discussion and Recommendations Regarding the Imaging Role of FAPI-Based PET

FAP-based imaging has brought about a significant shift in how we detect and evaluate tumors, thanks to its high specificity. The key to this lies in the overexpression of fibroblast activation protein (FAP) in cancer-associated fibroblasts (CAFs), which are found in nearly 90% of epithelial tumors. This makes FAP-specific inhibitors (FAPIs), when labeled with positron-emitting radionuclides, incredibly effective at zeroing in on both primary and metastatic tumors. What’s particularly exciting is how FAPI PET imaging stacks up against the conventional ^18^F-FDG PET/CT. Unlike FDG, which often struggles with high background activity in organs like the brain, heart, and liver, FAPI shows minimal uptake in these areas. This gives it a much-improved tumor-to-background ratio, allowing for clearer and more precise detection, especially in tumors with low glycolytic activity—think lobular breast cancer, mucinous tumors, and non-seminomatous germ cell tumors.

Another major plus is the favorable biodistribution and workflow that FAPI imaging offers. There’s no need for special patient preparation, and scans can begin as soon as 10 min after radiotracer injection. Its rapid clearance from non-target tissues not only enhances image contrast, but also cuts down scan time, making the whole process more efficient for both clinicians and patients. This efficiency, combined with its high sensitivity, has paved the way for FAPI PET imaging to be used across a wide range of oncological applications. In the central nervous system, for instance, FAPI PET/CT has proven to be superior in detecting brain tumors, particularly metastases, thanks to the negligible physiological brain uptake. Head and neck cancers, too, benefit from FAPI imaging’s enhanced ability to detect primary tumors and metastatic lymph nodes, even in cases where the primary tumor remains elusive with FDG PET/CT.

When it comes to thyroid, breast, and lung cancers, FAPI consistently shows a higher sensitivity and specificity, especially in tumor subtypes that FDG PET/CT struggles with. This extends to gastrointestinal and genitourinary malignancies, where FAPI has demonstrated an enhanced detection of primary tumors, lymph node involvement, and peritoneal metastases—an edge that is particularly valuable in cancers like gastric, pancreatic, colorectal, and bladder. One of the most promising aspects of FAPI imaging is its potential in image-guided therapy. Its high specificity for activated fibroblasts enables precise tumor delineation, which is crucial for targeted radiotherapy. Plus, the theranostic potential is compelling—imagine using the same molecule for both imaging and delivering therapeutic radionuclides, streamlining diagnosis and treatment into a single, cohesive approach.

But, as with any evolving technology, FAPI PET imaging comes with challenges. A significant issue is the occurrence of false positives due to FAPI uptake in non-malignant conditions. Fibrotic or inflammatory tissues—such as those seen in autoimmune diseases, benign tumors, or post-surgical scars—can also show tracer uptake, complicating image interpretation and potentially leading to overtreatment. Moreover, there is still a lack of standardized imaging protocols and interpretation criteria for FAPI PET/CT, which makes it difficult to reproduce results consistently across different centers and studies. Another hurdle is the variability in FAPI uptake among certain tumor types. Renal cell carcinoma and some subtypes of prostate cancer, for instance, exhibit inconsistent FAPI uptake, limiting its applicability in all cancer types. While FAPI PET/CT has demonstrated strong diagnostic capabilities, there are limited data on its effectiveness in longitudinal monitoring, particularly when it comes to tracking treatment response and guiding adaptive therapies over time.

Despite these challenges, the future of FAPI PET imaging looks bright. One promising development is its integration into multimodal imaging strategies. Combining FAPI PET/CT with other modalities like MRI could significantly enhance diagnostic accuracy, especially in complex cases like pancreatic cancer or brain tumors. There is also growing excitement around expanding the theranostic applications of FAPI. Early-phase studies are already exploring radiolabeled FAPIs for targeted radionuclide therapy, which could be a game changer for treating tumors that resist conventional therapies. Researchers are also working on developing more specific FAPI tracers to minimize non-specific uptake in benign tissues, which would help to reduce false positives and improve diagnostic precision.

Large-scale, multicenter trials are essential to validate FAPI PET/CT’s efficacy and safety across a broader range of cancer types. These studies will be critical in establishing standardized protocols and interpretation criteria, which will be key to integrating FAPI imaging into routine clinical practice. Looking ahead, FAPI PET/CT has the potential to play a pivotal role in personalized oncology. By providing detailed insights into the tumor microenvironment—particularly the extent of fibroblast activation—FAPI imaging could guide more tailored therapeutic approaches, ensuring that patients receive treatments specifically suited to their tumor biology.

## 10. FAPI-Based Theranostic Approaches

The summaries and evidence provided earlier in this chapter clearly illustrate the rapid expansion of FAPI-based PET applications in oncology. Even in areas such as cardiovascular disease, infection, and inflammatory conditions, its use has grown significantly, albeit with an expected delay in adoption. One of the key advantages of ^68^Ga-FAPI over FDG is its potential to bridge imaging with therapeutic applications. Given this, it is noteworthy that reports on FAPI-based therapies remain scarce, primarily limited to case studies and small patient series, with limited reproducibility (See Table 2 for an overview.)

A recent bibliometric analysis by Vd Hoven [118] identifies that, while FAPI theranostics is rapidly evolving, therapeutic applications remain underexplored, comprising only 5% of studies. Early investigations into radionuclide therapy with FAPI tracers, such as Lu-177 and Y-90, demonstrate promising tumor retention and acceptable toxicity profiles, but the field lacks randomized controlled trials (RCTs) to validate these findings [118].

Rezaei et al. [119] in their recent paper highlight the advancements in ^68^Ga-labeled FAPIs, particularly their enhanced biodistribution and tumor retention, which improve diagnostic accuracy. The development of dual-targeting radiotracers, like ^68^Ga-FAPI-RGD, shows the potential for superior tumor localization. Therapeutically, these tracers offer a foundation for combining diagnostic imaging with targeted radionuclide therapy, although challenges like nonspecific uptake persist [119].

Guglielmo et al. [50] highlighted FAPI-based theranostic approaches in thyroid cancer, where FAPI PET/CT surpasses ^18^FDG PET/CT in detecting metastases, especially in radioiodine-refractory differentiated thyroid cancer. Preliminary ^177^Lu-FAPI therapies have reported high response rates with minimal toxicity, though larger studies are needed to confirm these results [50].

Laudicella and colleagues [120] evaluated FAPI theranostics in prostate cancer, emphasizing its potential role in PSMA-negative and aggressive subtypes. FAPI-based radioligand therapy (RLT) using Lu-177 and Y-90 has shown feasibility and some efficacy, though issues like nonspecific uptake and a short tracer retention limit therapeutic outcomes.

A recent review by Baum and colleagues [33] reviewed the therapeutic landscape of FAPI, noting the success of FAPI tracers like ^68^Ga-FAPI-04 in imaging and their extension into therapy with isotopes such as Y-90, Lu-177, and Ac-225. Early-phase studies reveal a promising therapeutic efficacy, but rapid clearance from tumors remains a significant hurdle. Strategies like albumin-binding conjugates (e.g., ^177^Lu-EB-FAPI) aim to improve tumor retention and appear promising [33].

Langbein’s review [121] explores the broader context of theranostics, highlighting FAPI’s integration into precision oncology alongside established modalities like PRRT and PSMA-targeted therapy. Novel therapeutic targets and radioligands, including FAPI, show potential for expanding treatment options, though challenges like tracer clearance and optimized dosing regimens persist [121].

The systematic review “Fibroblast Activation Protein Inhibitor (FAPI)-Based Theranostics—Where We Are at and Where We Are Heading” consolidates these findings, emphasizing the promise of FAPI-based RLT. While initial results indicate a good tolerability and tumor control, heterogeneity in tracer design and pharmacokinetics complicates efficacy assessments [122]. The “Fibroblast Activation Protein-Based Theranostics in Cancer Research: A State-of-the-Art Review” reiterates FAPI’s therapeutic potential, focusing on strategies to improve tracer retention and reduce nonspecific uptake. It also discusses combining FAPI-based therapy with other modalities, such as immunotherapy, to enhance efficacy [48].

**Table 2:** Summarized details of the studies related to theranostics.

Author	Year of Publication	Country	Tracer	Population	Cancers
Baum et al. [123]	2022	Germany	^177^Lu-FAP-2286; ^68^Ga-FAPI-2286; ^68^Ga-FAPI-04	11 patients	5 pancreas; 4 breast; 1 rectum; 1 ovary.
Ferdinandus et al. [124]	2022	Germany	^90^Y-FAPI-46; ^68^Ga-FAPI-46	9 patients	3 pancreatic ductal adenocarcinoma; 4 sarcomas, 1 chordoma, 1 neuroendocrine tumor
Lindner et al. [34]	2020	Germany	^99m^Tc-FAP-34; ^68^Ga-FAPI-46; ^90^Y-FAPI-46	2 patients	1 pancreas; 1 ovarian
Lindner et al. [41]	2018	Germany	^90^Y-FAPI-04; ^68^Ga-FAPI-04	2 patients	2 breast
Ballal et al. [30]	2021	India	^177^Lu-DOTA.SA.FAPI; ^177^Lu-DOTAGA.(SA.FAPI)2 ^68^Ga-DOTA.SA.FAP	10 patients	5 thyroid; 4 breast; 1 paraganglioma
Ballal et al. [125]	2022	India	^68^Ga-DOTA.SA.FAPI; ^177^Lu-DOTAGA.(SA.FAPI)2	15 patients	15 thyroid cancers
Assadi et al. [126]	2021	Iran	^177^Lu-FAPI-46; ^68^Ga-FAPI-46	21 patients	2 ovarian cancer; 2 sarcomas, 3 colon cancer; 5 breast cancer; 2 pancreatic cancer; 2 prostate cancer; 1 cervical cancer; 1 lung cancer; 1 cholangiocarcinoma; 1 thyroid
Kuyumcu et al. [127]	2021	Turkey	^177^Lu-DOTA-FAPI-04; ^68^Ga-FAPI-04	4 patients	1 breast; 1 thymic carcinoma, 1 thyroid cancer, 1 ovarian cancer

## 11. Challenges and Limitations in FAPI Therapeutic Approaches

One of the primary obstacles in FAPI-based therapy lies in the complexity of the tumor microenvironment (TME). Cancer-associated fibroblasts (CAFs), which serve as the main targets for FAPI tracers, exhibit a dual role—contributing to tumor progression while also being involved in normal tissue repair and immune modulation. This dual functionality complicates selective targeting, as therapies must differentiate between malignant and physiological fibroblast activation to minimize adverse effects [119,122].

A further complicating factor is the heterogeneity in fibroblast activation protein (FAP) expression. This variability is observed not only between different tumor types, but also within individual tumors, leading to an inconsistent tracer uptake and response to therapy. Such heterogeneity poses challenges in patient selection and limits the broad applicability of FAPI-based approaches across diverse cancer subtypes [121,122].

Another concern is the potential for the non-specific uptake of FAPI tracers. While these tracers demonstrate a high affinity for FAP-expressing cells, they may also accumulate in areas of benign fibrosis or inflammation, leading to false-positive imaging results and possible off-target toxicities in therapeutic applications. Additionally, a background signal from healthy tissues exhibiting fibroblast activity can confound imaging interpretations and therapy planning [121].

Lastly, radiotracer stability and dosimetry optimization remain key technical hurdles. Ensuring the in vivo stability of FAPI radiotracers is crucial for accurate imaging and effective therapeutic delivery. Furthermore, optimizing dosimetry—particularly for therapeutic isotopes such as Lutetium-177 and Actinium-225—requires careful balancing to maximize tumor eradication while minimizing toxicity [33].

Despite promising preclinical data, the clinical evidence supporting FAPI theranostics remains limited. Much of the available data originate from case reports and small cohort studies, underscoring the urgent need for large-scale, prospective, and randomized clinical trials to establish its safety and therapeutic efficacy more definitively.

## 12. The Potential Role of FAPI PET-Based Theranostic Approaches

Despite the challenges, FAPI-based theranostics offer several unique advantages. Unlike conventional cancer imaging modalities that primarily target tumor cells, FAPI tracers target the tumor stroma, making them especially effective in tumors with dense stromal components, such as pancreatic, breast, and prostate cancers. This ability to visualize and target the tumor microenvironment provides an advantage over FDG-PET in cases where conventional imaging struggles with sensitivity. An additional advantage is FAPI’s potential role in PSMA-negative prostate cancer. While prostate-specific membrane antigen (PSMA) tracers have revolutionized prostate cancer imaging and therapy, a subset of patients with PSMA-negative disease remains underserved. FAPI offers a promising alternative, expanding personalized treatment strategies for prostate cancer patients who lack PSMA expression.

Beyond oncology, the role of FAPI in fibrotic diseases is an exciting area of exploration. Since FAP is implicated in fibrotic processes, FAPI tracers could be repurposed for diagnosing and potentially treating conditions such as cardiac and pulmonary fibrosis. This expands the clinical utility of FAPI theranostics into non-oncologic domains, fostering interdisciplinary research collaborations.

Another promising avenue is the integration of FAPI-based radionuclide therapy with other treatment modalities. Combination strategies involving chemotherapy, immunotherapy, or targeted agents may enhance overall treatment efficacy by disrupting the tumor stroma, potentially improving drug penetration and immune cell infiltration.

Finally, continued advancements in radiopharmaceutical development are expected to refine FAPI theranostics. Efforts to design FAPI ligands with an improved binding affinity, enhanced in vivo stability, and optimized pharmacokinetics are underway. Additionally, novel dual-modality imaging agents and alpha-emitting radiotherapeutics could further enhance both diagnostic precision and therapeutic impact.

In summary, FAPI theranostics holds significant therapeutic promise across various malignancies. While early clinical studies demonstrate encouraging results, ongoing research must address challenges like tracer pharmacokinetics and nonspecific uptake to fully realize FAPI’s potential in precision oncology. The development of fibroblast activation protein inhibitor (FAPI) theranostics presents both significant challenges and promising opportunities. A nuanced discussion of these factors is essential to advancing its clinical applications and optimizing its efficacy in oncologic and non-oncologic settings.

## 13. Conclusions

FAP-based PET imaging represents a significant advancement in oncologic and fibrotic disease imaging, offering superior tumor delineation, enhanced diagnostic accuracy, and the potential for targeted image-guided therapy. Its broad applicability across various malignancies and fibrotic conditions positions FAPI as a transformative tool in precision medicine. Several challenges remain, however, particularly regarding specificity, FAP heterogeneity, dosimetry optimization, and the establishment of standardized imaging protocols.

Future research efforts should focus on refining FAPI-based imaging agents to improve tumor selectivity, to minimize off-target uptake, and to optimize tracer kinetics for both diagnostic and therapeutic applications. Additionally, expanding clinical trials and fostering interdisciplinary collaborations will be critical in validating its role across diverse oncologic and non-oncologic conditions.

The integration of FAPI into theranostic strategies—leveraging both diagnostic imaging and targeted radionuclide therapy—holds immense promise for personalized treatment approaches. Advances in radiopharmaceutical development, improved tumor retention, and combination therapies may further enhance its clinical utility. As ongoing research continues to refine FAPI PET/CT applications, it is poised to become a cornerstone in the evolving landscape of nuclear medicine, offering new avenues for precision diagnosis and tailored therapy in cancer, cardiovascular disease, and inflammatory disorders.

## Figures and Tables

**Table 1:** Overview of the major FAP ligands.

FAP Ligand	Diagnostic Isotope	Therapeutic Isotope	Reference	Comment
FAP-02	^68^Ga		Lindner et al. [41]	Early small-molecule FAP tracer
FAP-04	^68^Ga	^90^Y, ^225^Ac	Lindner et al. [41], Watabe et al. [42]	First clinical applied small-molecule FAP tracer
FAP-34	^99m^Tc	^188^Re	Lindner et al. [34]	Applied for diagnostic scintigraphy and SPECT, theranostic application
Tc99m FAPI	^99m^Tc		Wang et al. [45]	Applied for diagnostic scintigraphy and SPECT
FAP-46	^68^Ga	^90^Y, ^153^Sm	Loktev et al. [43], Kratochwil et al. [44]	Applied for diagnostic, potential theranostic application
FAP-74	^68^Ga, ^18^F		Giesel et al. [26], Lindner et al. [27]	Applied for diagnostic PET with ^18^F, potential application with ^68^Ga
OncoFAP-DOTAGA	^68^Ga		Backhaus et al. [28], Millul et al. [29]	Applied for diagnostic PET, considered for theranostic application
DOTA.SA.FAPI	^68^Ga		Ballal et al. [30]	Applied for diagnostic PET
Boron pro-alanine-FAPI	^68^Ga		Poplawski et al. [19]	Applied for diagnostic PET
DOTAGA. (SA.FAPI)_2_	^68^Ga	^177^Lu	Ballal et al. [30]	Applied for diagnostic PET, theranostic application

## Data Availability

No new data were created or analyzed in this study. Data sharing is not applicable to this article.

## References

[1] Balkwill F.R., Capasso M., Hagemann T. (2012). The tumor microenvironment at a glance. J. Cell Sci..

[2] Zhang Y., Weinberg R.A. (2018). Epithelial-to-mesenchymal transition in cancer: Complexity and opportunities EMT: A naturally occurring transdifferentiation program. Front. Med..

[3] Busek P., Mateu R., Zubal M., Kotackova L., Sedo A. (2018). Targeting fibroblast activation protein in cancer—Prospects and caveats. Front. Biosci. (Landmark Ed.).

[4] Baghban R., Roshangar L., Jahanban-Esfahlan R., Seidi K., Ebrahimi-Kalan A., Jaymand M., Kolahian S., Javaheri T., Zare P. (2020). Tumor microenvironment complexity and therapeutic implications at a glance. Cell Commun. Signal..

[5] Kratochwil C., Flechsig P., Lindner T., Abderrahim L., Altmann A., Mier W., Adeberg S., Rathke H., Röhrich M., Winter H. (2019). ^68^Ga-FAPI PET/CT: Tracer uptake in 28 different kinds of cancer. J. Nucl. Med..

[6] Giesel F.L., Kratochwil C., Lindner T., Marschalek M.M., Loktev A., Lehnert W., Debus J., Jäger D., Flechsig P., Altmann A. (2019). ^68^Ga-FAPI PET/CT: Biodistribution and preliminary dosimetry estimate of 2 DOTA-containing FAP-targeting agents in patients with various cancers. J. Nucl. Med..

[7] Loktev A., Lindner T., Mier W., Debus J., Altmann A., Jäger D., Giesel F., Kratochwil C., Barthe P., Roumestand C. (2018). A tumor imaging method targeting cancer-associated fibroblasts. J. Nucl. Med..

[8] Jacobson F.L., Van den Abbeele A.D. (2022). Importance of ^68^Ga-FAPI PET/CT for detection of cancer. Radiology.

[9] Lind P., Igerc I., Beyer T., Reinprecht P., Hausegger K. (2004). Advantages and limitations of FDG PET in the follow-up of breast cancer. Eur. J. Nucl. Med. Mol. Imaging.

[10] Hess S., Scholtens A.M., Gormsen L.C. (2020). Patient preparation and patient-related challenges with FDG-PET/CT in infectious and inflammatory disease. PET Clin..

[11] Mori Y., Novruzov E., Schmitt D., Cardinale J., Watabe T., Choyke P.L., Alavi A., Haberkorn U., Giesel F.L. (2024). Clinical applications of fibroblast activation protein inhibitor positron emission tomography (FAPI-PET). NPJ Imaging.

[12] Peterson C., Denlinger N., Yang Y. (2022). Recent advances and challenges in cancer immunotherapy. Cancers.

[13] Fitzgerald A.A., Weiner L.M. (2020). The role of fibroblast activation protein in health and malignancy. Cancer Metastasis Rev..

[14] Rettig W.J., Garin-Chesa P., Beresford H.R., Oettgen H.F., Melamed M.R., Old L.J. (1988). Cell-surface glycoproteins of human sarcomas: Differential expression in normal and malignant tissues and cultured cells. Proc. Natl. Acad. Sci. USA.

[15] Chandekar K.R., Prashanth A., Vinjamuri S., Kumar R. (2023). FAPI PET/CT Imaging-An Updated Review. Diagnostics.

[16] Ballal S., Yadav M.P., Moon E.S., Kramer V.S., Roesch F., Kumari S., Tripathi M., ArunRaj S.T., Sarswat S., Bal C. (2021). Biodistribution, Pharmacokinetics, Dosimetry of [^68^Ga]Ga-DOTA.SA.FAPi, and the Head-to-Head Comparison with [^18^F]F-FDG PET/CT in Patients with Various Cancers. Eur. J. Nucl. Med. Mol. Imaging.

[17] Chen H., Zhao L., Ruan D., Pang Y., Hao B., Dai Y., Wu X., Guo W., Fan C., Wu J. (2021). Usefulness of [^68^Ga]Ga-DOTA-FAPI-04 PET/CT in patients presenting with inconclusive [^18^F]FDG PET/CT findings. Eur. J. Nucl. Med. Mol. Imaging.

[18] Lan L., Liu H., Wang Y., Deng J., Peng D., Feng Y., Wang L., Chen Y., Qiu L. (2022). The potential utility of [^68^Ga]Ga-DOTA-FAPI-04 as a novel broad-spectrum oncological and non-oncological imaging agent-comparison with [^18^F]FDG. Eur. J. Nucl. Med. Mol. Imaging.

[19] Poplawski S.E., Lai J.H., Li Y., Jin Z., Liu Y., Wu W., Wu Y., Zhou Y., Sudmeier J.L., Sanford D.G. (2013). Identification of selective and potent inhibitors of fibroblast activation protein and prolyl oligopeptidase. J. Med. Chem..

[20] Linz C., Brands R.C., Kertels O., Dierks A., Brumberg J., Gerhard-Hartmann E., Hartmann S., Schirbel A., Serfling S., Zhi Y. (2021). Targeting fibroblast activation protein in newly diagnosed squamous cell carcinoma of the oral cavity: Initial experience and comparison to [18F]FDG PET/CT and MRI. Eur. J. Nucl. Med. Mol. Imaging.

[21] Guo W., Pang Y., Yao L., Zhao L., Fan C., Ke J., Guo P., Hao B., Fu H., Xie C. (2021). Imaging fibroblast activation protein in liver cancer: A single-center post hoc retrospective analysis to compare [^68^Ga]Ga-FAPI-04 PET/CT versus MRI and [^18^F]-FDG PET/CT. Eur. J. Nucl. Med. Mol. Imaging.

[22] Sollini M., Kirienko M., Gelardi F., Fiz F., Gozzi N., Chiti A. (2021). State-of-the-art of FAPI-PET imaging: A systematic review and meta-analysis. Eur. J. Nucl. Med. Mol. Imaging.

[23] Roustaei H., Kiamanesh Z., Askari E., Sadeghi R., Aryana K., Treglia G. (2022). Could fibroblast activation protein (FAP)-specific radioligands be considered as pan-tumor agents?. Contrast Media Mol. Imaging.

[24] Gege Z., Xueju W., Bin J. (2023). Head-to-head comparison of ^68^Ga-FAPI PET/CT and FDG PET/CT for the detection of peritoneal metastases: Systematic review and meta-analysis. Am. J. Roentgenol..

[25] Huang D., Wu J., Zhong H., Li Y., Han Y., He Y., Chen Y., Lin S., Pang H. (2023). [^68^Ga]Ga-FAPI PET for the evaluation of digestive system tumors: Systematic review and meta-analysis. Eur. J. Nucl. Med. Mol. Imaging.

[26] Giesel F.L., Adeberg S., Syed M., Lindner T., Jiménez-Franco L.D., Mavriopoulou E., Staudinger F., Tonndorf-Martini E., Regnery S., Rieken S. (2021). FAPI-74 PET/CT using either ^18^F-AlF or cold-kit ^68^Ga labeling: Biodistribution, radiation dosimetry, and tumor delineation in lung cancer patients. J. Nucl. Med..

[27] Lindner T., Altmann A., Giesel F., Kratochwil C., Kleist C., Krämer S., Mier W., Cardinale J., Kauczor H.U., Jäger D. (2021). ^18^F-labeled tracers targeting fibroblast activation protein. EJNMMI Radiopharm. Chem..

[28] Backhaus P., Gierse F., Burg M.C., Büther F., Asmus I., Dorten P., Cufe J., Roll W., Neri D., Cazzamalli S. (2022). Translational imaging of the fibroblast activation protein (FAP) using the new ligand, [^68^Ga]Ga-OncoFAP-DOTAGA. Eur. J. Nucl. Med. Mol. Imaging.

[29] Millul J., Bassi G., Mock J., Elsayed A., Pellegrino C., Zana A., Dakhel Plaza S., Nadal L., Gloger A., Schmidt E. (2021). An ultra-high-affinity small organic ligand of fibroblast activation protein for tumor-targeting applications. Proc. Natl. Acad. Sci. USA.

[30] Ballal S., Yadav M.P., Moon E.S., Kramer V.S., Roesch F., Kumari S., Bal C. (2021). First-In-Human Results on the Biodistribution, Pharmacokinetics, and Dosimetry of [^177^Lu]Lu-DOTA.SA.FAPi and [^177^Lu]Lu-DOTAGA.(SA.FAPi)_2_. Pharmaceuticals.

[31] Chen H., Pang Y., Li J., Kang F., Xu W., Meng T., Shang Q., Zhao J., Guan Y., Wu H. (2023). Comparison of [^68^Ga]Ga-FAPI and [^18^F]FDG uptake in patients with gastric signet-ring-cell carcinoma: A multicenter retrospective study. Eur. Radiol..

[32] Röhrich M., Naumann P., Giesel F.L., Choyke P.L., Staudinger F., Wefers A., Liew D.P., Kratochwil C., Rathke H., Liermann J. (2021). Impact of ^68^Ga-FAPIPET/CT imaging on the therapeutic management of primary and recurrent pancreatic ductal adenocarcinomas. J. Nucl. Med..

[33] Baum R.P., Novruzov E., Zhao T., Greifenstein L., Jakobsson V., Perrone E., Mishra A., Eismant A., Ghai K., Klein O. (2024). Radiomolecular theranostics with fibroblast-activation-protein inhibitors and peptides. Semin. Nucl. Med..

[34] Lindner T., Altmann A., Krämer S., Kleist C., Loktev A., Kratochwil C., Giesel F., Mier W., Marme F., Debus J. (2020). Design and Development of ^99m^Tc-Labeled FAPI Tracers for SPECT Imaging and ^188^Re Therapy. J. Nucl. Med..

[35] Mori Y., Haberkorn U., Giesel F.L. (2023). ^68^Ga- or 18F-FAPI PET/CT—What it can and cannot. Eur. Radiol..

[36] Hamson E.J., Keane F.M., Tholen S., Schilling O., Gorrell M.D. (2014). Understanding fibroblast activation protein (FAP): Substrates, activities, expression and targeting for cancer therapy. Proteom. Clin. Appl..

[37] Chen X., Song E. (2019). Turning foes to friends: Targeting cancer-associated fibroblasts. Nat. Rev. Drug Discov..

[38] Dong Y., Zhou H., Alhaskawi A., Wang Z., Lai J., Yao C., Liu Z., Ezzi S.H.A., Kota V.G., Abdulla M.H.A.H. (2023). The Superiority of Fibroblast Activation Protein Inhibitor (FAPI) PET/CT Versus FDG PET/CT in the Diagnosis of Various Malignancies. Cancers.

[39] Lopci E., Fanti S. (2020). Non-FDG PET/CT. Recent Results Cancer Res..

[40] Wass G., Clifford K., Subramaniam R.M. (2023). Evaluation of the Diagnostic Accuracy of FAPI PET/CT in Oncologic Studies: Systematic Review and Metaanalysis. J. Nucl. Med..

[41] Lindner T., Loktev A., Altmann A., Giesel F., Kratochwil C., Debus J., Jäger D., Mier W., Haberkorn U. (2018). Development of quinoline-based theranostic ligands for the targeting of fibroblast activation protein. J. Nucl. Med..

[42] Watabe T., Liu Y., Kaneda-Nakashima K., Shirakami Y., Lindner T., Ooe K., Toyoshima A., Nagata K., Shimosegawa E., Haberkorn U. (2020). Theranostics Targeting Fibroblast Activation Protein in the Tumor Stroma: 64Cu- and 225Ac-Labeled FAPI-04 in Pancreatic Cancer Xenograft Mouse Models. J. Nucl. Med..

[43] Loktev A., Lindner T., Burger E.M., Altmann A., Giesel F., Kratochwil C., Debus J., Marmé F., Jäger D., Mier W. (2019). Development of Fibroblast Activation Protein-Targeted Radiotracers with Improved Tumor Retention. J. Nucl. Med..

[44] Kratochwil C., Giesel F.L., Rathke H., Fink R., Dendl K., Debus J., Mier W., Jäger D., Lindner T., Haberkorn U. (2021). [^153^Sm]Samarium-labeled FAPI-46 radioligand therapy in a patient with lung metastases of a sarcoma. Eur. J. Nucl. Med. Mol. Imaging.

[45] Wang Y., Gu C., Gai Y., Hu J., Sun X. (2024). The ^99m^Tc-labeled FAPI peptide probes exhibit excellent targeting specificity and sensitivity to FAP in SPECT/CT imaging. J. Nucl. Med..

[46] Liu Y., Ding H., Cao J., Liu G., Chen Y., Huang Z. (2024). [^68^Ga] Ga-FAPI PET/CT in brain tumors: Comparison with [^18^F]F-FDG PET/CT. Front. Oncol..

[47] Hua T., Chen M., Fu P., Zhou W., Zhao W., Li M., Zuo C., Guan Y., Xu H. (2024). Heterogeneity of fibroblast activation protein expression in the microenvironment of an intracranial tumor cohort: Head-to-head comparison of gallium-68 FAP inhibitor-04 (^68^Ga-FAPi-04) and fluoride-18 fluoroethyl-L-tyrosine (^18^F-FET) in positron emission tomography-computed tomography imaging. Quant. Imaging Med. Surg..

[48] Zhao L., Chen J., Pang Y., Fu K., Shang Q., Wu H., Sun L., Lin Q., Chen H. (2022). Fibroblast activation protein-based theranostics in cancer research: A state-of-the-art review. Theranostics.

[49] Croce L., Teliti M., Chytiris S., Sparano C., Coperchini F., Villani L., Calì B., Petrone L., Magri F., Trimboli P. (2024). The American Thyroid Association risk classification of papillary thyroid cancer according to presurgery cytology. Eur. J. Endocrinol..

[50] Guglielmo P., Alongi P., Baratto L., Conte M., Abenavoli E.M., Buschiazzo A., Celesti G., Dondi F., Filice R., Gorica J. (2024). FAPi-based agents in thyroid cancer: A new step towards diagnosis and therapy? A systematic review of the literature. Cancers.

[51] Ballal S., Yadav M.P., Satapathy S., Roesch F., Chandekar K.R., Martin M., Shakir M., Agarwal S., Rastogi S., Moon E.S. (2025). Long-Term Outcomes in Radioiodine-Resistant Follicular Cell-Derived Thyroid Cancers Treated with [^177^Lu]Lu-DOTAGA.FAPi Dimer Therapy. Thyroid.

[52] Nourbakhsh S., Salehi Y., Farzanehfar S., Ghaletaki R., Kashi M.B., Abbasi M. (2024). FAPI PET/CT provides higher uptake and better target to back ground in recurrent and metastatic tumors of patients with Iodine refractory papillary thyroid cancer compared with FDG PET CT. Nuklearmedizin.

[53] Wang M., Liu H., Zhang J., Wu B., Zhang C. (2024). Progress in the application of radiolabeled FAPI in advanced differentiated thyroid cancer. Clin. Transl. Imaging.

[54] Zhang J., Xiong J., Wang M., Wu B., Zhang C. (2024). Comparison of the diagnostic value of ^68^Ga-FAPI and 18F-FDG PET/CT in breast cancer: A systematic review. Clin. Transl. Imaging.

[55] Elboga U., Sahin E., Kus T., Cayirli Y.B., Aktas G., Uzun E., Cinkir H.Y., Teker F., Sever O.N., Aytekin A. (2021). Superiority of ^68^Ga-FAPI PET/CT scan in detecting additional lesions compared to ^18^FDG PET/CT scan in breast cancer. Ann. Nucl. Med..

[56] Guo W., Xu W., Meng T., Fan C., Fu H., Pang Y., Zhao L., Sun L., Huang J., Mi Y. (2025). FAP-targeted PET/CT imaging in patients with breast cancer from a prospective bi-center study: Insights into diagnosis and clinic management. Eur. J. Nucl. Med. Mol. Imaging.

[57] Chen L., Zheng S., Chen L., Xu S., Wu K., Kong L., Xue J., Chen X., Miao W., Zhu Y. (2023). ^68^Ga-labeled fibroblast activation protein inhibitor PET/CT for the early and late prediction of pathologic response to neoadjuvant chemotherapy in breast cancer patients: A prospective study. J. Nucl. Med..

[58] Novruzov E., Mori Y., Alavi A., Giesel F.L. (2024). The impact of FAP imaging in lung cancer and beyond: A new chapter. Eur. Radiol..

[59] Yang Q., Huang D., Wu J., Zhong H., Han Y., Jiang H., Chen Y., Chen G., Zhan X., Zhou P. (2024). Performance of [^18^F] FDG PET/CT versus FAPI PET/CT for lung cancer assessment: A systematic review and meta-analysis. Eur. Radiol..

[60] Wan Q.C., Bai L., Wang Z.Y., Ji B. (2024). Diagnostic performance of FAPI PET/CT for the detection of lymph node metastases in lung cancer patients: A meta-analysis. Acad. Radiol..

[61] Li Y., Zhang Y., Guo Z., Hou P., Lv J., Ke M., Liu S., Li S., Yin W., He J. (2024). [^18^F] FAPI adds value to [^18^F] FDG PET/CT for diagnosing lymph node metastases in stage I-IIIA non-small cell lung cancer: A prospective study. Cancer Imaging.

[62] Zhao L., Chen S., Chen S., Pang Y., Dai Y., Hu S., Lin L.E., Fu L., Sun L., Wu H. (2021). ^68^Ga-fibroblast activation protein inhibitor PET/CT on gross tumour volume delineation for radiotherapy planning of oesophageal cancer. Radiother. Oncol..

[63] Liu H., Yang X., You Z., Hu Z., Chen Y. (2023). Role of ^68^Ga-FAPI-04 PET/CT in the initial staging of Esophageal Cancer. Nuklearmedizin.

[64] Ristau J., Giesel F.L., Haefner M.F., Staudinger F., Lindner T., Merkel A., Schlittenhardt J., Kratochwil C., Choyke P.L., Herfarth K. (2020). Impact of primary staging with fibroblast activation protein specific enzyme inhibitor (FAPI)-PET/CT on radio-oncologic treatment planning of patients with esophageal cancer. Mol. Imaging Biol..

[65] Wegen S., Claus K., Linde P., Rosenbrock J., Trommer M., Zander T., Tuchscherer A., Bruns C., Schlößer H.A., Schröder W. (2024). Impact of FAPI-46/dual-tracer PET/CT imaging on radiotherapeutic management in esophageal cancer. Radiat. Oncol..

[66] Öztürk A.A., Flamen P. (2023). FAP-targeted PET imaging in gastrointestinal malignancies: A comprehensive review. Cancer Imaging.

[67] Xie W., Li B., Hong Z., Zhang Y. (2024). Head-to-head comparison of [^18^F] FDG PET and [^68^Ga] Ga-FAPI-04 PET in the diagnosis of gastric and pancreatic cancer: A systematic review and meta-analysis. Clin. Transl. Imaging.

[68] Wu Q., Wang C., Huang C., Li D. (2024). Head-to-head comparison of [^68^Ga]Ga-FAPI-04 PET and [^18^F]FDG PET in the detection of cancer recurrence: A systematic review and meta-analysis. Transl. Cancer Res..

[69] Hu X., Li X., Wang P., Cai J. (2024). The Role of FAPI PET Imaging in Pancreatic Cancer: A Meta-analysis Compared with ^18^F-FDG PET. Acad. Radiol..

[70] Tang R., Liu M., Shu Q., Chen X., Cai L. (2024). Performance of fibroblast activating protein inhibitor PET imaging for pancreatic neoplasms assessment: A systematic review and meta-analysis. Eur. Radiol..

[71] Singh P., Singhal T., Parida G.K., Rahman A., Agrawal K. (2024). Diagnostic performance of FAPI PET/CT vs. 18F-FDG PET/CT in evaluation of liver tumors: A systematic review and Meta-analysis. Mol. Imaging Radionucl. Ther..

[72] Manuppella F., Pisano G., Taralli S., Caldarella C., Calcagni M.L. (2024). Diagnostic performances of PET/CT using fibroblast activation protein inhibitors in patients with primary and metastatic liver tumors: A Comprehensive Literature Review. Int. J. Mol. Sci..

[73] Zhuang Z., Zhang Y., Yang X., Deng X., Wang Z. (2024). Head-to-head comparison of the diagnostic performance between ^68^Ga-FAPI-04 PET/CT and ^18^F-FDG PET/CT in colorectal cancer: A systematic review and meta-analysis. Abdom. Radiol..

[74] Lin X., Li Y., Wang S., Zhang Y., Chen X., Wei M., Zhu H., Wu A., Yang Z., Wang X. (2023). Diagnostic value of [^68^Ga]Ga-FAPI-04 in patients with colorectal cancer in comparison with [^18^F]F-FDG PET/CT. Front. Oncol..

[75] Sun L., Hao P., Peng R. (2024). Comparison of ^68^Ga-FAPI PET CT/MRI and ^18^F-FDG PET/CT in metastatic lesions of gynecological cancers: A systematic review and head-to-head meta-analysis. Acta Radiol..

[76] Dendl K., Koerber S.A., Finck R., Mokoala K.M., Staudinger F., Schillings L., Heger U., Röhrich M., Kratochwil C., Sathekge M. (2021). ^68^Ga-FAPI-PET/CT in patients with various gynecological malignancies. Eur. J. Nucl. Med. Mol. Imaging.

[77] Shu Q., He X., Chen X., Liu M., Chen Y., Cai L. (2023). Head-to-head comparison of ^18^F-FDG and ^68^Ga-FAPI-04 PET/CT for radiological evaluation of cervical cancer. Clin. Nucl. Med..

[78] Gu B., Liu X., Wang S., Xu X., Liu X., Hu S., Yan W., Luo Z., Song S. (2022). Head-to-head evaluation of [^18^F]FDG and [^6^Ga]Ga-DOTA-FAPI-04 PET/CT in recurrent soft tissue sarcoma. Eur. J. Nucl. Med. Mol. Imaging.

[79] Wu C., Wen F., Lin F., Zeng Y., Lin X., Hu X., Zhang X., Zhang X., Wang X. (2024). Predictive performance of [^18^F]F-fibroblast activation protein inhibitor (FAPI)-42 positron emission tomography/computed tomography (PET/CT) in evaluating response of recurrent or metastatic gastrointestinal stromal tumors: Complementary or alternative to [^18^F]fluorodeoxyglucose (FDG) PET/CT?. Quant. Imaging Med. Surg..

[80] Ortolan N., Urso L., Zamberlan I., Filippi L., Buffi N.M., Cittanti C., Uccelli L., Bartolomei M., Evangelista L. (2024). Is there a role for FAPI PET in urological cancers?. Mol. Diagn. Ther..

[81] Hagens M.J., van Leeuwen P.J., Wondergem M., Boellaard T.N., Sanguedolce F., Oprea-Lager D.E., Bex A., Vis A.N., van der Poel H.G., Mertens L.S. (2024). A Systematic Review on the Diagnostic Value of Fibroblast Activation Protein Inhibitor PET/CT in Genitourinary Cancers. J. Nucl. Med..

[82] Wynn T.A. (2004). Fibrotic disease and the T_H_1/T_H_2 paradigm. Nat. Rev. Immunol..

[83] Nagaraju C.K., Dries E., Popovic N., Singh A.A., Haemers P., Roderick H.L., Claus P., Sipido K.R., Driesen R.B. (2017). Global fibroblast activation throughout the left ventricle but localized fibrosis after myocardial infarction. Sci. Rep..

[84] Loganath K., Craig N., Barton A., Joshi S., Anagnostopoulos C., Erba P.A., Glaudemans A.W., Saraste A., Bucerius J., Lubberink M. (2024). Cardiovascular PET imaging of fibroblast activation: A review of the current literature. J. Nucl. Cardiol..

[85] Mewton N., Liu C.Y., Croisille P., Bluemke D., Lima J.A.C. (2011). Assessment of Myocardial Fibrosis with Cardiovascular Magnetic Resonance. J. Am. Coll. Cardiol..

[86] Lee I.K., Noguera-Ortega E., Xiao Z., Todd L., Scholler J., Song D., Liousia M., Lohith K., Xu K., Edwards K.J. (2022). Monitoring Therapeutic Response to Anti-FAP CAR T Cells Using [^18^F]AlF-FAPI-74. Clin. Cancer Res..

[87] Lyu Z., Han W., Zhao H., Jiao Y., Xu P., Wang Y., Shen Q., Yang S., Zhao C., Tian L. (2022). A clinical study on relationship between visualization of cardiac fibroblast activation protein activity by Al^18^F-NOTA-FAPI-04 positron emission tomography and cardiovascular disease. Front. Cardiovasc. Med..

[88] Higuchi T., Serfling S.E., Leistner D.M., Speer T., Werner R.A. (2024). FAPI-PET in Cardiovascular Disease. Semin. Nucl. Med..

[89] Fan D., Takawale A., Lee J., Kassiri Z. (2012). Cardiac fibroblasts, fibrosis and extracellular matrix remodelling in heart disease. Fibrogenesis Tissue Repair.

[90] Tillmanns J., Hoffmann D., Habbaba Y., Schmitto J.D., Sedding D., Fraccarollo D., Galuppo P., Bauersachs J. (2015). Fibroblast activation protein alpha expression identifies activated fibroblasts after myocardial infarction. J. Mol. Cell. Cardiol..

[91] Varasteh Z., Mohanta S., Robu S., Braeuer M., Li Y., Omidvari N., Topping G., Sun T., Nekolla S.G., Richter A. (2019). Molecular Imaging of Fibroblast Activity After Myocardial Infarction Using a ^68^Ga-Labeled Fibroblast Activation Protein Inhibitor, FAPI-04. J. Nucl. Med..

[92] Diekmann J., Koenig T., Thackeray J.T., Derlin T., Czerner C., Neuser J., Ross T.L., Schäfer A., Tillmanns J., Bauersachs J. (2022). Cardiac Fibroblast Activation in Patients Early After Acute Myocardial Infarction: Integration with MR Tissue Characterization and Subsequent Functional Outcome. J. Nucl. Med..

[93] Xie B., Wang J., Xi X.Y., Guo X., Chen B.X., Li L., Hua C., Zhao S., Su P., Chen M. (2022). Fibroblast activation protein imaging in reperfused ST-elevation myocardial infarction: Comparison with cardiac magnetic resonance imaging. Eur. J. Nucl. Med. Mol. Imaging.

[94] Sun F., Wang C., Du X. (2022). [^18^F]AlF-NOTA-FAPI-04 PET imaging of fibroblast activation protein in heart failure with preserved ejection fraction. J. Nucl. Med..

[95] Song W., Zhang X., He S., Gai Y., Qin C., Hu F., Wang Y., Wang Z., Bai P., Wang J. (2023). ^68^Ga-FAPI PET visualize heart failure: From mechanism to clinic. Eur. J. Nucl. Med. Mol. Imaging.

[96] Wei Z., Xu H., Chen B., Wang J., Yang X., Yang M.F., Zhao S. (2024). Early detection of anthracyclineinduced cardiotoxicity using [^68^Ga]Ga-FAPI-04 imaging. Eur. J. Nucl. Med. Mol. Imaging.

[97] Wang L., Wang Y., Wang J., Xiao M., Xi X.Y., Chen B.X., Su Y., Zhang Y., Xie B., Dong Z. (2022). Myocardial Activity at ^18^FFAPI PET/CT and Risk for Sudden Cardiac Death in Hypertrophic Cardiomyopathy. Radiology.

[98] Puls M., Beuthner B.E., Topci R., Vogelgesang A., Bleckmann A., Sitte M., Lange T., Backhaus S.J., Schuster A., Seidler T. (2020). Impact of myocardial fibrosis on left ventricular remodelling, recovery, and outcome after transcatheter aortic valve implantation in different haemodynamic subtypes of severe aortic stenosis. Eur. Heart J..

[99] Diekmann J., Neuser J., Röhrich M., Derlin T., Zwadlo C., Koenig T., Weiberg D., Jäckle F., Kempf T., Ross T.L. (2023). Molecular Imaging of Myocardial Fibroblast Activation in Patients with Advanced Aortic Stenosis Before Transcatheter Aortic Valve Replacement: A Pilot Study. J. Nucl. Med..

[100] Monslow J., Todd L., Chojnowski J.E., Govindaraju P.K., Assoian R.K., Puré E. (2020). Fibroblast activation protein regulates lesion burden and the fibroinflammatory response in Apoe-deficient mice in a sexually dimorphic manner. Am. J. Pathol..

[101] Dendl K., Koerber S.A., Finck R., Mokoala K.M., Staudinger F., Schillings L., Heger U., Röhrich M., Kratochwil C., Sathekge M. (2019). Vascular smooth muscle cells in atherosclerosis. Nat. Rev. Cardiol..

[102] Kosmala A., Serfling S.E., Michalski K., Lindner T., Schirbel A., Higuchi T., Hartrampf P.E., Derlin T., Buck A.K., Weich A. (2023). Molecular imaging of arterial fibroblast activation protein: Association with calcified plaque burden and cardiovascular risk factors. Eur. J. Nucl. Med. Mol. Imaging.

[103] Wu M., Ning J., Li J., Lai Z., Shi X., Xing H., Hacker M., Liu B., Huo L., Li X. (2022). Feasibility of In Vivo Imaging of Fibroblast Activation Protein in Human Arterial Walls. J. Nucl. Med..

[104] Wu S., Pang Y., Zhao L., Zhao L., Chen H. (2021). ^68^Ga-FAPI PET/CT Versus 18F-FDG PET/CT for the Evaluation of Disease Activity in Takayasu Arteritis. Clin. Nucl. Med..

[105] Cho E., Park C.H., Kim J., Kim K., Kim S.S. (2023). Serial ^68^Ga-FAPI PET/CT After Treatment of Immunoglobulin G4-Related Pancreatitis and Retroperitoneal Fibrosis. Clin. Nucl. Med..

[106] Lartey D.A., Schilder L.A., Zwezerijnen G.J.C., D’haens G.R.A.M., Grootjans J., Löwenberg M. (2025). FAPi PET/CT Imaging to Identify Fibrosis in Immune-Mediated Inflammatory Diseases. Biomedicines.

[107] Zhong K., Chen H., Hou P., Cheng L., Guo W., Li Y., Lv J., Ke M., Wu X., Lei Y. (2025). Comparison of [^18^F]FAPI-42 and [^18^F]FDG PET/CT in the evaluation of systemic vasculitis. Eur. J. Nucl. Med. Mol. Imaging.

[108] Schmidkonz C., Kuwert T., Götz T.I., Ramming A., Atzinger A. (2024). Recent advances in nuclear medicine and their role in inflammatory arthritis: Focus on the emerging role of FAPI PET/CT. Skelet. Radiol..

[109] Hotta M., Kim G.H., Rerkpichaisuth V., Teng P.Y., Armstrong W.R., Carlucci G., Dahlbom M., Abtin F., Lari S.M., Fishbein G.A. (2024). Correlation of FAPI PET uptake with immunohistochemistry in explanted lungs from patients with advanced interstitial lung disease. J. Nucl. Med..

[110] Hao B., Wu X., Pang Y., Sun L., Wu H., Huang W., Chen H. (2021). [^18^F]FDG and [^68^Ga]Ga-DOTA-FAPI-04 PET/CT in the evaluation of tuberculous lesions. Eur. J. Nucl. Med. Mol. Imaging.

[111] Liu W., Gong W., Yang X., Xu T., Chen Y. (2023). Increased FAPI activity in pulmonary tuberculosis. Clin. Nucl. Med..

[112] Sviridenko A., Boehm A., di Santo G., Uprimny C., Nilica B., Fritz J., Giesel F.L., Haberkorn U., Sahanic S., Decristoforo C. (2022). Enhancing Clinical Diagnosis for Patients with Persistent Pulmonary Abnormalities After COVID-19 Infection: The Potential Benefit of ^68^Ga-FAPI PET/CT. Clin. Nucl. Med..

[113] Musameh K., O’Brien S., Mehboob R., Butler T., Cunningham Z., Buckley C., Atzinger A., Kuwert T., Mitchell P., Donnelly S.C. (2024). Evaluation of fibroblast activation protein-specific PET/CT in a patient with post-COVID pneumonitis. Respirol. Case Rep..

[114] Kullik Y., Wessendorf T.E., Theegarten D., Winantea J., Hautzel H., Opitz M. (2024). *Aspergillus fumigatus*: Is Dual-Tracer ^18^FDG/^68^Ga-FAPI PET/CT Capable of Distinguishing Fungal Infection and Unspecific Inflammation from Recurrent Lung Cancer?. Clin. Nucl. Med..

[115] Wang Y., Wang R., Zhang X., Li L., Liu H., Chang Y., Li Q., Wang Y., Qi E., Hao L. (2023). Diagnostic performance of [^68^Ga]Ga-DOTA-FAPI-04 for periprosthetic hip joint infection. Eur. J. Nucl. Med. Mol. Imaging.

[116] Bentestuen M., Al-Obaydi N., Zacho H.D. (2023). FAPI-avid nonmalignant PET/CT findings: An expedited systematic review. Semin. Nucl. Med..

[117] Albano D., Rizzo A., Slart R.H., Hess S., Noriega-Álvarez E., Wakfie-Corieh C.G., Leccisotti L., Glaudemans A.W., Gheysens O., Treglia G. (2024). The Role of Fibroblast Activation Protein Inhibitor Positron Emission Tomography in Inflammatory and Infectious Diseases: An Updated Systematic Review. Pharmaceuticals.

[118] Hoven A.F.v.D., Keijsers R.G.M., Lam M.G.E.H., Glaudemans A.W.J.M., Verburg F.A., Vogel W.V., Lavalaye J. (2023). Current research topics in FAPI theranostics: A bibliometric analysis. Eur. J. Nucl. Med. Mol. Imaging.

[119] Rezaei S., Gharapapagh E., Dabiri S., Heidari P., Aghanejad A. (2023). Theranostics in targeting fibroblast activation protein bearing cells: Progress and challenges. Life Sci..

[120] Laudicella R., Spataro A., Crocè L., Giacoppo G., Romano D., Davì V., Lopes M., Librando M., Nicocia A., Rappazzo A. (2023). Preliminary findings of the role of FAPi in prostate cancer theranostics. Diagnostics.

[121] Langbein T., Weber W.A., Eiber M. (2019). Future of theranostics: An outlook on precision oncology in nuclear medicine. J. Nucl. Med..

[122] Sidrak M.M.A., De Feo M.S., Corica F., Gorica J., Conte M., Filippi L., Schillaci O., De Vincentis G., Frantellizzi V. (2023). Fibroblast activation protein inhibitor (FAPI)-based theranostics—Where we are at and where we are heading: A systematic review. Int. J. Mol. Sci..

[123] Baum R.P., Schuchardt C., Singh A., Chantadisai M., Robiller F.C., Zhang J., Mueller D., Eismant A., Almaguel F., Zboralski D. (2022). Feasibility, Biodistribution, and Preliminary Dosimetry in Peptide-Targeted Radionuclide Therapy of Diverse Adenocarcinomas Using ^177^Lu-FAP-2286: First-in-Humans Results. J. Nucl. Med..

[124] Ferdinandus J., Costa P.F., Kessler L., Weber M., Hirmas N., Kostbade K., Bauer S., Schuler M., Ahrens M., Schildhaus H.U. (2022). Initial Clinical Experience with ^90^Y-FAPI-46 Radioligand Therapy for Advanced-Stage Solid Tumors: A Case Series of 9 Patients. J. Nucl. Med..

[125] Ballal S., Yadav M.P., Moon E.S., Roesch F., Kumari S., Agarwal S., Tripathi M., Sahoo R.K., Mangu B.S., Tupalli A. (2022). Novel Fibroblast Activation Protein Inhibitor-Based Targeted Theranostics for Radioiodine-Refractory Differentiated Thyroid Cancer Patients: A Pilot Study. Thyroid.

[126] Assadi M., Rekabpour S.J., Jafari E., Divband G., Nikkholgh B., Amini H., Kamali H., Ebrahimi S., Shakibazad N., Jokar N. (2021). Feasibility and Therapeutic Potential of ^177^Lu-Fibroblast Activation Protein Inhibitor-46 for Patients With Relapsed or Refractory Cancers: A Preliminary Study. Clin. Nucl. Med..

[127] Kuyumcu S., Kovan B., Sanli Y., Buyukkaya F., Has Simsek D., Özkan Z.G., Isik E.G., Ekenel M., Turkmen C. (2021). Safety of Fibroblast Activation Protein-Targeted Radionuclide Therapy by a Low-Dose Dosimetric Approach Using ^177^Lu-FAPI04. Clin. Nucl. Med..

